# Disease activity in primary progressive multiple sclerosis: a systematic review and meta-analysis

**DOI:** 10.3389/fneur.2023.1277477

**Published:** 2023-11-06

**Authors:** Katelijn M. Blok, Joost van Rosmalen, Nura Tebayna, Joost Smolders, Beatrijs Wokke, Janet de Beukelaar

**Affiliations:** ^1^Department of Neurology, Albert Schweitzer Hospital, Dordrecht, Netherlands; ^2^Department of Neurology, MS Center ErasMS, Erasmus University Medical Center, Rotterdam, Netherlands; ^3^Department of Biostatistics, Erasmus University Medical Center, Rotterdam, Netherlands; ^4^Department of Epidemiology, Erasmus University Medical Center, Rotterdam, Netherlands; ^5^Department of Immunology, MS Center ErasMS, Erasmus University Medical Center, Rotterdam, Netherlands; ^6^Neuroimmunology Researchgroup, Netherlands Institute for Neuroscience, Amsterdam, Netherlands

**Keywords:** primary progressive multiple sclerosis, disease activity, systematic review, multiple sclerosis, inflammation

## Abstract

**Background:**

Disease activity in multiple sclerosis (MS) is defined as presence of relapses, gadolinium enhancing lesions and/or new or enlarging lesions on MRI. It is associated with efficacy of immunomodulating therapies (IMTs) in primary progressive MS (PPMS). However, a thorough review on disease activity in PPMS is lacking. In relapsing remitting MS, the prevalence of activity decreases in more contemporary cohorts. For PPMS, this is unknown.

**Aim:**

To review disease activity in PPMS cohorts and identify its predictors.

**Methods:**

A systematic search in EMBASE, MEDLINE, Web of science Core Collection, COCHRANE CENTRAL register of trials, and GOOGLE SCHOLAR was performed. Keywords included PPMS, inflammation, and synonyms. We included original studies with predefined available data, extracted cohort characteristics and disease activity outcomes and performed meta-regression analyses.

**Results:**

We included 34 articles describing 7,109 people with PPMS (pwPPMS). The weighted estimated proportion of pwPPMS with overall disease activity was 26.8% (95% CI 20.6–34.0%). A lower age at inclusion predicted higher disease activity (OR 0.91, *p* = 0.031). Radiological activity (31.9%) was more frequent than relapses (9.2%), and was predicted by longer follow-up duration (OR 1.27, *p* = 0.033). Year of publication was not correlated with disease activity.

**Conclusion:**

Inflammatory disease activity is common in PPMS and has remained stable over the last decades. Age and follow-up duration predict disease activity, advocating prolonged monitoring of young pwPPMS to evaluate potential IMT benefits.

## 1. Introduction

The phenotype definitions of multiple sclerosis (MS) (1) distinguish between three disease courses (relapsing remitting (RRMS), secondary progressive (SPMS), and primary progressive MS (PPMS)) and add temporal information about two main disease processes: disease activity and disease progression (1). Disease activity is thought to reflect active inflammatory processes with focal disruption of the blood brain barrier (2), and is defined as the presence of at least one of the following: (i) clinical relapses, (ii) occurrence of gadolinium enhancing T1 lesions (GEL) or new or unequivocally enlarging T2 hyperintense lesions (NEL) (1). Disease progression is thought to reflect neurodegenerative processes and/or compartmentalized chronic inflammation (2), and is defined as a steadily increasing objectively documented neurologic dysfunction without unequivocal recovery and independent of relapse activity (1).

Insight into these disease processes in people with MS (pwMS) is important, because most current MS treatments are immunomodulating therapies (IMTs) that aim at reducing disease activity (3). Also in progressive MS, there is evidence from observational studies (4) and from (subgroup analyses of) randomized controlled trials with anti-CD20 therapies or siponimod (5–7) that inflammatory disease activity is modifiable by IMTs, with some effect on long-term disability accumulation. Therefore, thorough assessment of disease activity in progressive MS is warranted in order to judge whether pwMS may benefit from IMT. Recent efforts have been made to gain insight into disease activity in SPMS (8, 9), but for PPMS this is still lacking. Of late there have been observations that study populations of people with RRMS have changed over time. More recent RRMS cohorts show lower rates of disease activity (10, 11), milder disease courses (10, 12), and older ages at diagnosis (13, 14). It is unknown if such changes have also occurred in the PPMS population, which may affect its eligibility for IMTs. Therefore, in this systematic review we aimed to get an overview of disease activity in PPMS as reported in literature and to identify its predictors. Additionally, we investigated whether PPMS study populations have changed over time.

## 2. Methods

We followed the updated Preferred Reporting Items for Systematic Reviews and Meta-analyses (PRISMA) (15) guideline (Supplementary S1).

### 2.1. Literature search

A systematic literature search was conducted by an information specialist. Databases included EMBASE, MEDLINE, Web of science Core Collection, COCHRANE CENTRAL register of trials, and GOOGLE SCHOLAR. We used no restrictions to language, publication type or date. We searched the databases from inception until 07 June 2021. Several synonyms for PPMS and inflammatory activity were used (Supplementary S2). Title/abstract screening was performed with EndNote (16). All abstracts were screened in DUPLO by three authors (KB, NT, and JB) on available data on clinical or radiological signs of disease activity in a minimum of 5 people with PPMS (pwPPMS). Grey literature was excluded. After abstract screening, all full text articles were assessed by two authors (KB and JB) on:

- Full-text availability in English;
- Original research paper;
- Minimum of 5 adult pwPPMS (to avoid exclusion of relevant studies with low sample size, while maintaining a lower limit for validity of the data);
- Sufficient information about PPMS-cohort: available data on age and definition of PPMS;
- Sufficient information about disease activity: available data on relapses, GEL, and/or NEL (as number of cases and sample size or percentage);
- Cohort not already included in other article, to prevent bias towards multiply described cohorts. The article containing the most data was included.

In case of disagreement of eligibility between the first two raters, articles were discussed with a third rater (BW), after which inclusion was based on agreement. After this procedure, references of included articles were screened for potential additional eligible articles.

### 2.2. Data collection

From included articles, data were extracted by one person (KB) using a prespecified spreadsheet. The following data were extracted:

- Predictors: number of pwPPMS, diagnostic criteria used, midpoint age/disease duration/EDSS (all at study inclusion), percentage of female patients, number of pwPPMS on IMT, duration of follow-up and for radiological disease activity the number of MRIs and the dose of gadolinium used (because of possible influence on number of GEL found) (17). The number of pwPPMS on IMT was recorded for the relevant outcome: in case of a disease activity outcome at baseline (i.e., cases of GEL at baseline), the number of pwPPMS on IMT within the 3 months before baseline were recorded, but for a disease activity outcome at follow-up (i.e., cases of NEL or relapses) the number of pwPPMS on IMT during follow-up was recorded. The number of pwPPMS on IMT could therefore be 0% for outcomes in a placebo group.
- Outcomes on disease activity data: cases of pwPPMS with GEL, NEL and/or relapses. For radiological disease activity, both brain and spinal cord MRI were taken into account.

Because the included articles used different units to represent their data, we adhered to the following definitions: the ‘midpoint’ of a variable (e.g., age or disease duration) was defined as the mean, or median if the mean was unavailable. If no median or mean of follow-up duration was available but >70% completed a certain duration of follow-up, then that time was chosen as median follow-up. For cross-sectional studies and for outcomes only reported at baseline, a follow-up of 0.01 years was recorded.

Corresponding authors from two articles were contacted with questions regarding the described PPMS cohorts. Because a wide variety of study designs could be included, methodological quality was assessed using the Mixed Methods Appraisal Tool (MMAT) (18). No restrictions to study quality were applied for inclusion in our meta-analyses.

### 2.3. Statistical analyses

The prevalence of disease activity (GEL, NEL and/or relapses) from each study was calculated using raw data (i.e., number of cases divided by sample size). If only percentages were given, the number of cases was back calculated from the reported sample size.

To better approximate a normal distribution for meta-analyses and meta-regression, prevalence rates were transformed with a logit (log odds) transformation (19). For studies reporting zero activity outcomes, we used a standard bias/continuity correction of adding *n* = 0.5 to the number of cases and increasing the sample size by *n* = 1 before logit transformation. We calculated the estimated pooled prevalence of disease activity outcomes with a random-effects model meta-analysis using the logit transformed data, and then back-transformed the estimate. The variance between studies was estimated with a restricted maximum likelihood method. The amount of heterogeneity was reported using the *I*^2^-statistic. We performed meta-regression analyses to explore causes of the heterogeneity in reported disease activity outcomes. Univariable and multivariable models were fitted for each of the disease activity outcome measures:

- Clinical activity: percentage of pwPPMS with relapses;
- Radiological activity: percentage of pwPPMS with GEL and/or NEL;
- Overall disease activity: the maximum percentage of pwPPMS with relapses, GEL or NEL. For instance, if an article reported that 5% of pwPPMS experienced relapses, 10% of pwPPMS had GEL at baseline but 30% had NEL on follow-up MRI, then an overall disease activity of 30% was recorded.

For multivariable analyses, we pre-specified four clinically relevant variables: midpoint age, EDSS, and disease duration at inclusion, and follow-up time of study. Other variables were added if they showed a *p* < 0.2 in univariable analyses and were available from at least 10 studies. We chose to enter year of publication into the models instead of diagnostic criteria used, because we could not evenly transform the diagnostic criteria into a continuous variable for this model, and there were too many missing data for years of inclusion for the described cohorts. We performed a sensitivity analysis for the multivariable meta-regression by using a beta-binomial model with a logit link function as alternative model. Finally, as an *ad-hoc* analysis, a Mann–Whitney U test was performed to compare the midpoint age at inclusion between randomized controlled trials (RCTs) of IMTs with a positive and negative outcome on disease progression.

We analyzed any correlation between cohort characteristics and year of publication with Spearman’s rank correlation tests.

Statistical analyses were performed in IBM SPSS Statistics 28, with the exception of the beta-binomial model which was performed in R, using the package VGAM (20). All analyses were performed with a two-sided significance level of 0.05.

### 2.4. Publication bias

Publication bias was not evaluated in this review because we did not focus on study endpoints such as positive/negative results of drug efficacy, but on cohort characteristics of pwPPMS, for which no publication bias was assumed. For the same reason, no data on funding sources of studies were collected.

## 3. Results

### 3.1. Included articles

**Figure 1:**
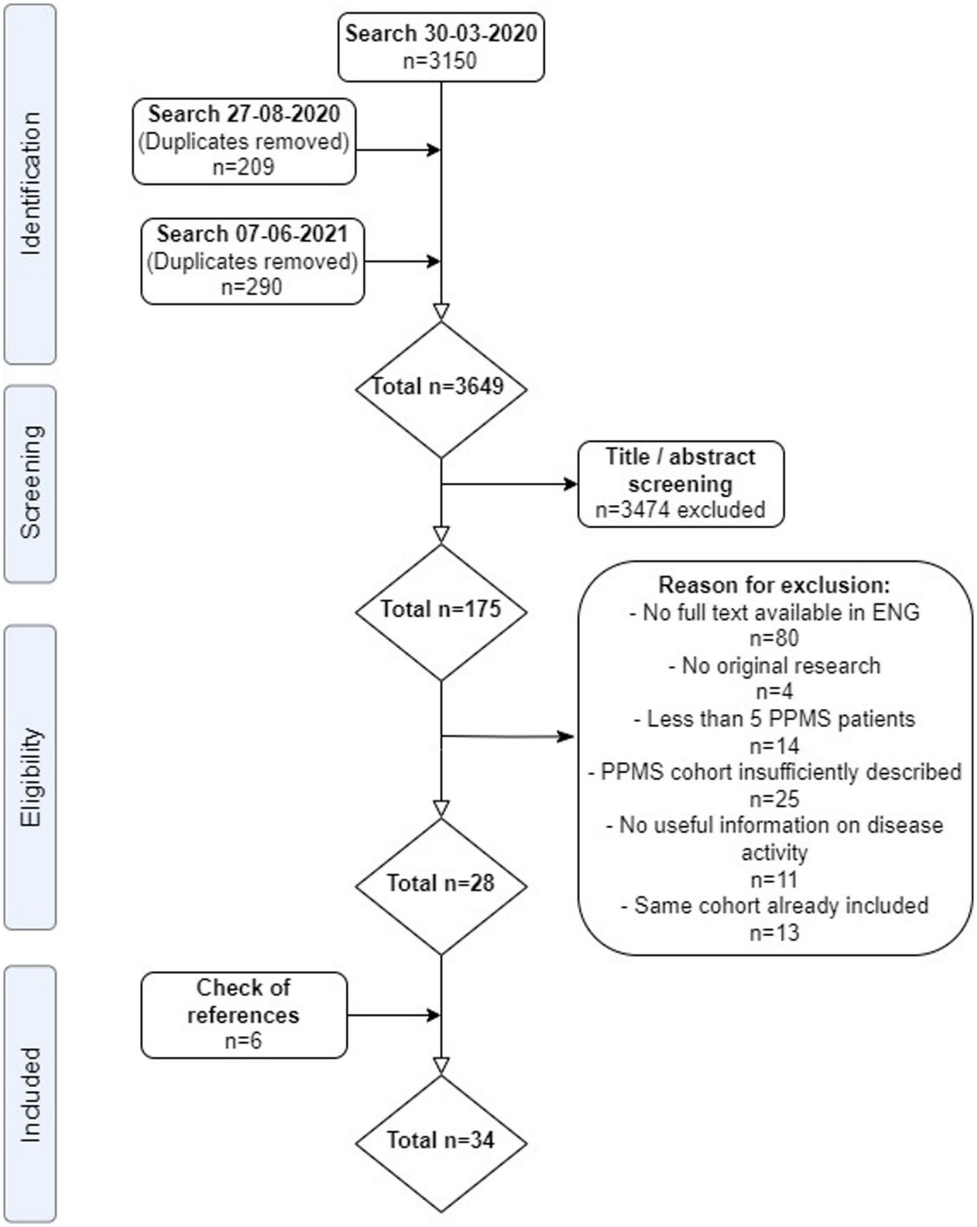
PRISMA flow-chart of systematic review. ENG, English; PPMS, primary progressive multiple sclerosis. From: Moher et al. (21).

A total of 3,649 non-duplicate articles were identified. After screening on title/abstract, 175 articles were assessed in full text for eligibility, including screening of references. We included 28 articles from our search, plus another 6 articles after screening references, adding to a total of 34 included articles (Figure 1) (5, 6, 17, 22–52). Reasons for exclusion after full text assessment are given in the Supplementary Data S3; apart from unavailable full text versions, the most common reason for exclusion was insufficiently described PPMS cohort characteristics. Results of the study appraisals can be found in Supplementary S4. In general, the quality of included studies was good. The study by Araujo et al. (22) showed a high risk of bias, with a non-representative sample of pwPPMS and a non-standardized follow-up, possibly influencing disease activity outcomes in this (small) cohort. The study by Lorscheider et al. (33) showed risk of bias for interpreting the treatment efficacy, but because from this study we only recorded the baseline (pre-treatment) activity data, this possible bias was irrelevant for our analyses. Study characteristics of included articles are shown in Table 1.

**Table 1:** Overview of included articles.

Study	Study characteristics						Inclusion criteria							Quality
	Design and location	I.P.	F/N	Inclusion period	Outcome activity	% on IMT	Age	Diagnostic criteria	Disease activity	Positive CSF***	Medication use	Disease duration	EDSS	MMAT score
Araujo 2008	Non-RCT. Uni. SM-Am.	IVMP	7/11*	1975–1993	REL	100%	N.A.	MD’01, MD’05	N.A.	N.A.	N.A.	N.A.	N.A.	0/5
Beutler 1996	RCT. Uni. N-Am.	I.v. CLA	34/51	1992-N.A	REL, GEL	0%	N.A.	Poser	N.A.	N.A.	N.A.	>2 years	N.A.	4/5
Calabrese 2012	DIA. Uni. Eur.		26/44	2005–2006	REL, GEL, NEL	56.8%	N.A.	MD’01	No relapse ≤6 m	N.A.	N.A.	N.A.	N.A.	5/5
Disano 2020	ETI. Uni. N-Am.		4/13		Overall	38.5%	18–75	MD’17	N.A.	N.A.	No CS ≤ 30 d	N.A.	N.A.	3/5
Donninelli 2021	ETI. Multi. Eur.		14/26		GEL	0%	N.A.	MD’10	N.A.	Yes	No DMT	N.A.	N.A.	5/5
Fernandez-Diaz 2020	Non-RCT. Multi. Eur.	OCR	25/59	N.A.-2020	REL, GEL, NEL	22.0%	N.A.	MD’17	N.A.	N.A.	≥1 OCR infusion	N.A.	N.A.	3/5
Filippi 1995	DIA. Uni. Eur.		N.A./10*		GEL	N.A.	N.A.	N.A.	N.A.	N.A.	N.A.	N.A.	N.A.	4/5
Giovannoni 2020	RCT. Multi. Eur, N-Am.	LAQ	169/374*	2015–2016	GEL, NEL	0%	25–55	MD’10	No relapse ever	N.A.	N.A.	N.A.	3.0–6.5	5/5
Harding 2015	NAT. Uni. Eur.		121/234*	1985-N.A.	REL	N.A.	N.A.	MD’10	N.A.	N.A.	N.A.	N.A.	N.A.	4/5
Hawker 2009	RCT. Multi. N-Am.	RIT	221/439*		GEL, NEL	5.0%	18–65	MD‘01	No relapse ever	Yes	No ‘recent’ DMT	≤1 year	2.0–6.5	4/5
Hughes 2018	NAT. Multi. WW.		773/1419*	1995–2017	REL	30.4%	≥18	MD’05, MD’10	N.A.	No	N.A.	N.A.	N.A.	4/5
Khaleeli 2010	DIA. Uni. Eur.		17/45*		GEL	4.4%	N.A.	Th2000	N.A.	N.A.	N.A.	≤5 years	N.A.	4/5
Leary 2003	RCT. Uni. Eur.	IFN	18/50*		REL	0%	18–60	N.A	No relapse ever	Yes	No DMT or CS ≤ 2 m	≥2 years	2.0–7.0	4/5
Lorscheider 2019	Non-RCT. Multi. WW.	Any DMT	282/533*	1995–2018	REL, GEL, NEL	36.6%	N.A.	MD’05, MD’10	N.A.	No	No IMT before first visit	N.A.	N.A.	2/5
Lublin 2016	RCT. Multi. Eur, N-Am.	FIN	395/823*	2008–2011	REL, GEL, NEL	0%	25–65	MD’05	No relapse ever	No	No CS or DMT ≤ 3 m, no IST ≤ 7 m, no MT ≤ 5 y, no CLA ever	2–10 years	3.5–6.0	4/5
Montalban 2017	RCT. Multi. WW.	OCR	361/732*	2011–2012	REL, GEL, NEL	0%	18–55	MD’05	No ‘PRMS’	Yes	No BCT, ALE, CLA, IST, NAT ever. No INF, GA ≤ 12 w. No CS ≤ 4 w	≤15 y if EDSS > 5.0, or ≤10 y if EDSS ≤ 5.0	3.0–6.5	5/5
Naser Moghadasi 2019	Non-RCT. Uni. Asia.	RIT	8/20		REL, GEL, NEL	N.A.	N.A.	MD’10	N.A.	No	≥1 infusion of RIT ≤ 6 m	≥2 years	N.A.	3/5
Perez-Miralles 2021	NAT. Multi. Eur		24/55*	2017	REL, GEL, NEL	0%	≥18	MD’10	N.A.	N.A.	No DMT ≤ 6 m	<10 years	N.A.	4/5
Petrou 2020	RCT. Uni. Asia.	AMCST (IV or IT)	0/7	2015–2018	REL, GEL, NEL	100%	18–65	Poser	Other**	N.A.	At least 1 prior DMT (failure)	N.A.	3.0–6.5	4/5
Pohlau 2007	RCT. Multi. Eur.	IVIG	13/34	1997–2000	REL	50%	18–65	Poser	No relapse ≤12 m	No	No CS ≤ 1 m. No IMT ≤ 3 m	≥2 years	3.0–7.0	4/5
Ratzer 2016	Non-RCT. Uni. Eur.	OMP	11/15	2011–2012	REL	0%	18–65	N.A.	No relapse ≤1 m	No	No CS ≤ 3 m. No INF, GA ≤ 3 m. No IST ≤ 6 m	N.A.	<7.0	3/5

Study	Study characteristics						Inclusion criteria							Quality
	Design and location	I.P.	F/N	Study	Outcome activity	% on IMT	Age	Diagnostic criteria	Disease activity	Positive CSF***	Medication use	Disease duration	EDSS	Design
Romme-Christensen 2014	Non-RCT. Uni. Eur.	NAT	6/12	2010–2012	REL, GEL, NEL	0%	18–65	MD’05	No relapse ≤1 m	No	No CS or IMT ≤ 3 m. No IST ≤ 6 m	N.A.	<7.0	3/5
Salzer 2016	Non-RCT. Multi. Eur.	RIT	32/67	2001–2015	GEL	47.8%	N.A.	N-A	N.A.	No	≥1 infusion of RIT	N.A.	N.A.	3/5
Sastre-Garriga 2005	PRO. Uni. Eur.		13/31*		GEL	0%	N.A.	Th2000	N.A.	No	N.A.	<5 years	N.A.	5/5
Thompson 1991	DIA. Uni. Eur.		N.A./12		GEL, NEL	N.A.	N.A.	Poser	No relapse ever	Yes	N.A.	N.A.	N.A.	4/5
Wolinsky 2007	RCT. Multi. Eur, N-Am.	GA	483/943*	1999–2000	GEL, NEL	0%	30–65	N-A	No relapse ever	No	No INF, IST, IMT, CS ≤ 3 m	N.A.	3.0–6.5	4/5
Zephir 2004	Non-RCT. Multi. Eur.	CYC	61/128		REL	100%	N.A.	Poser	N.A.	No	N.A.	N.A.	N.A.	4/5
Zecca 2020	Non-RCT. Multi. Eur.	RIT	N.A./43	2009–2019	GEL, NEL	67.4%	N.A.	MD’17	N.A.	No	Treated with RIT	N.A.	N.A.	4/5
Confavreux 2006	NAT. Uni. Eur.		161/282	1957–1997	REL	0%	N.A.	Poser	N.A.	No	N.A.	N.A.	N.A.	4/5
Lycklama a Nijeholt 1998	DIA. Uni. Eur.		17/31		GEL	N.A.	N.A.	Poser	N.A.	No	N.A.	N.A.	N.A.	5/5
Mateo Paz Soldan 2014	NAT. Uni. N-Am.		174/322	1992-N.A.	REL	27.0%	≥18	MD’05	N.A.	No	N.A.	N.A.	N.A.	4/5
Kidd 1996	DIA. Uni. Eur.		N.A./10		GEL, NEL	N.A.	N.A.	Poser	No relapse ever	Yes	N.A.	N.A.	N.A.	4/5
Kremenchutzky 1999	NAT. Uni. N-Am.		124/218	1972–1984	REL	N.A.	N.A.	N-A	N.A.	No	N.A.	N.A.	N.A.	4/5
Silver 1997	DIA. Uni. Eur.		N.A./16		GEL	0%	24–56	Poser	No relapse ≤1 m	No	No CS, IMT ≤ 6 m	N.A.	N.A.	3/5

RCT, randomized controlled trial; non-RCT, therapeutic study other than RCT; ETI, etiological study; DIA, diagnostic study; PRO, prognostic study; NAT, natural history study; Uni, unicenter; Multi, multicenter; Eur, Europe; N-Am, North-America; SM-Am, South and/or Middle America; WW, worldwide (more than 2 regions); I.P., investigational product; GEL, gadolinium enhancing lesions; NEL, new or enlarging lesions; REL, relapses; CSF, cerebrospinal fluid; CS, corticosteroids; IFN, interferons ß-1a; GA, glatiramer acetate; DMT, disease modifying treatment; IMT, immunomodulating therapy; MX, Mitoxatrone; CLA, cladribine; ALE, alemtuzumab; IST, immune suppressive treatment; BCT, B-cell treatment; NAT, natalizumab; FIN, fingolimod; RIT, rituximab; OCR, ocrelizumab; IVMP, intravenous methylprednisolon; LAQ, laquinimod; AMCST, autologous mesenchymal stem cell transplantation; IVIG, intravenous immunoglobulin; OMP, oral methylprednisolone; CYC, cyclophosphamide; MD, McDonald criteria; Th, Thompson criteria; N.A., not available; EDSS, expanded disability status scale; MMAT, mixed methods appraisal tool (scale of 0–5); F/N, number of female/total people with PPMS in study. *Only PPMS (otherwise: subgroup of larger cohort), **active or worsening progressive MS (according to Lublin) and treatment failure of ≥1 DMT as evidenced either ≥2 relapses, new MRI activity or by deterioration in EDSS, and ***defined as presence of unique oligoclonal bands in CSF and/or elevated IgG-index.

**Figure 2:**
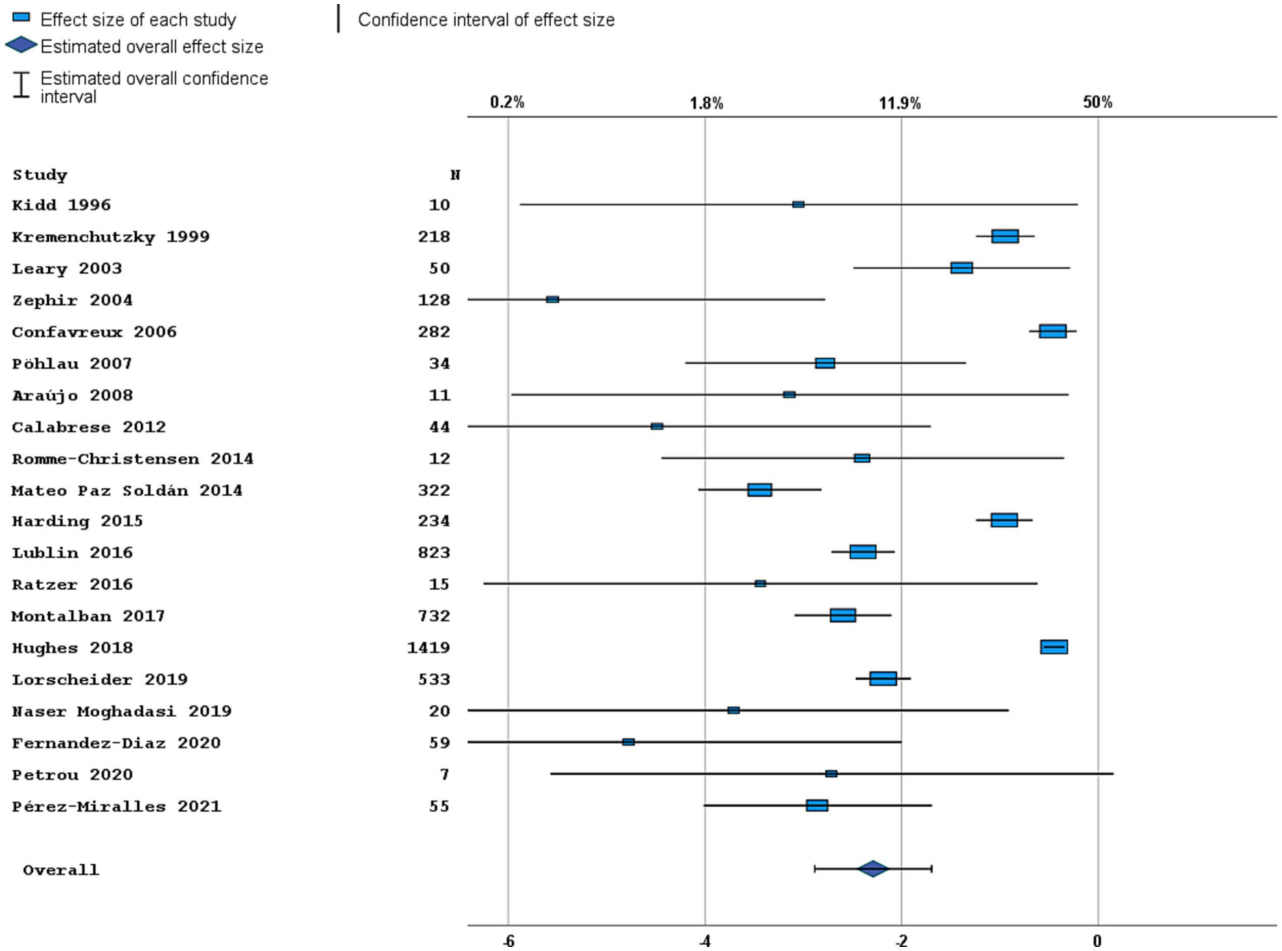
Forest plot of clinical disease activity outcome. Random effects model. Lower axis shows logit scale, upper axis shows prevalence. *N*, number of total included people with PPMS.

**Figure 3:**
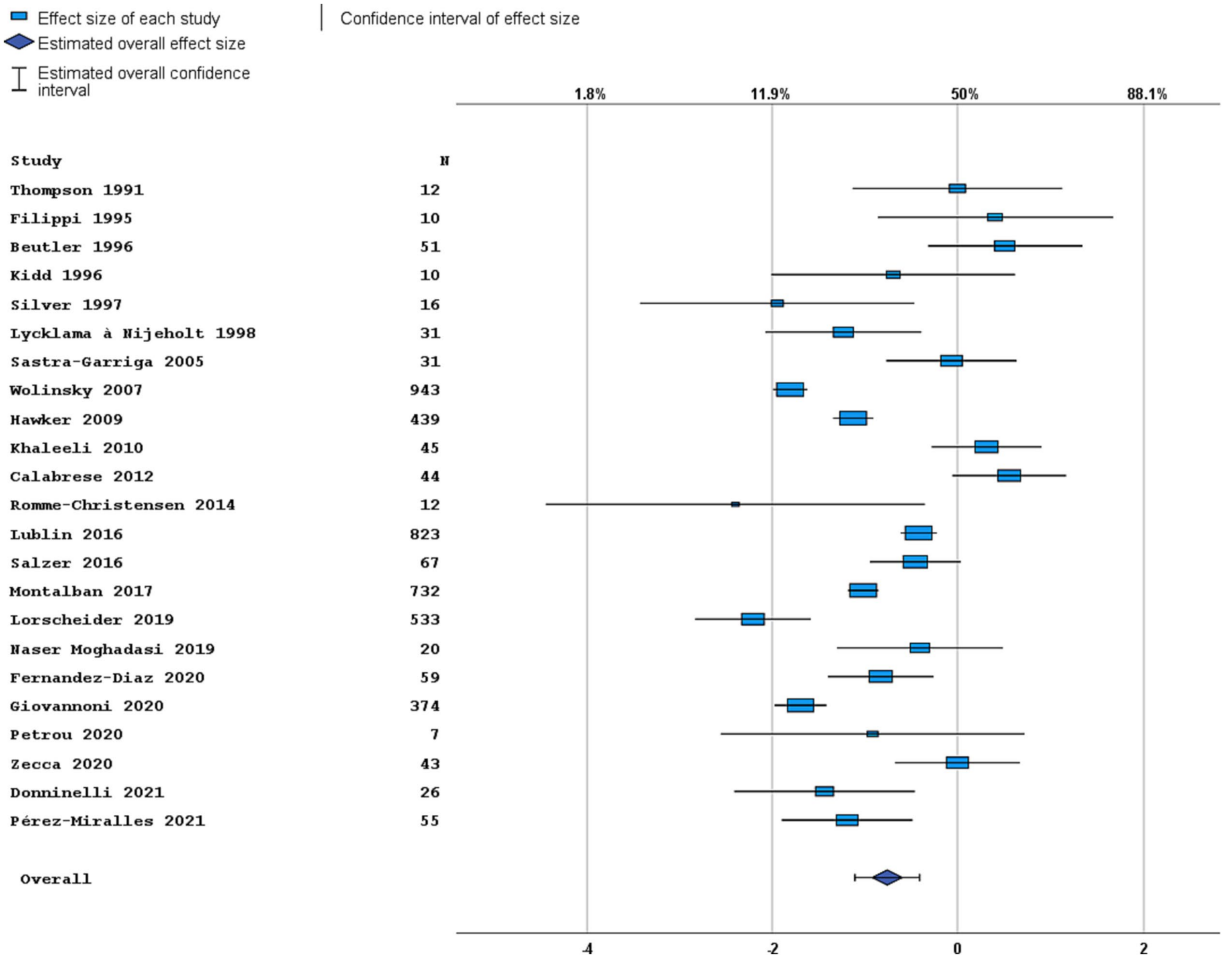
Forest plot of radiological disease activity outcome. Random effects model. Lower axis shows logit scale, upper axis shows prevalence. *N*, number of total included people with PPMS.

**Figure 4:**
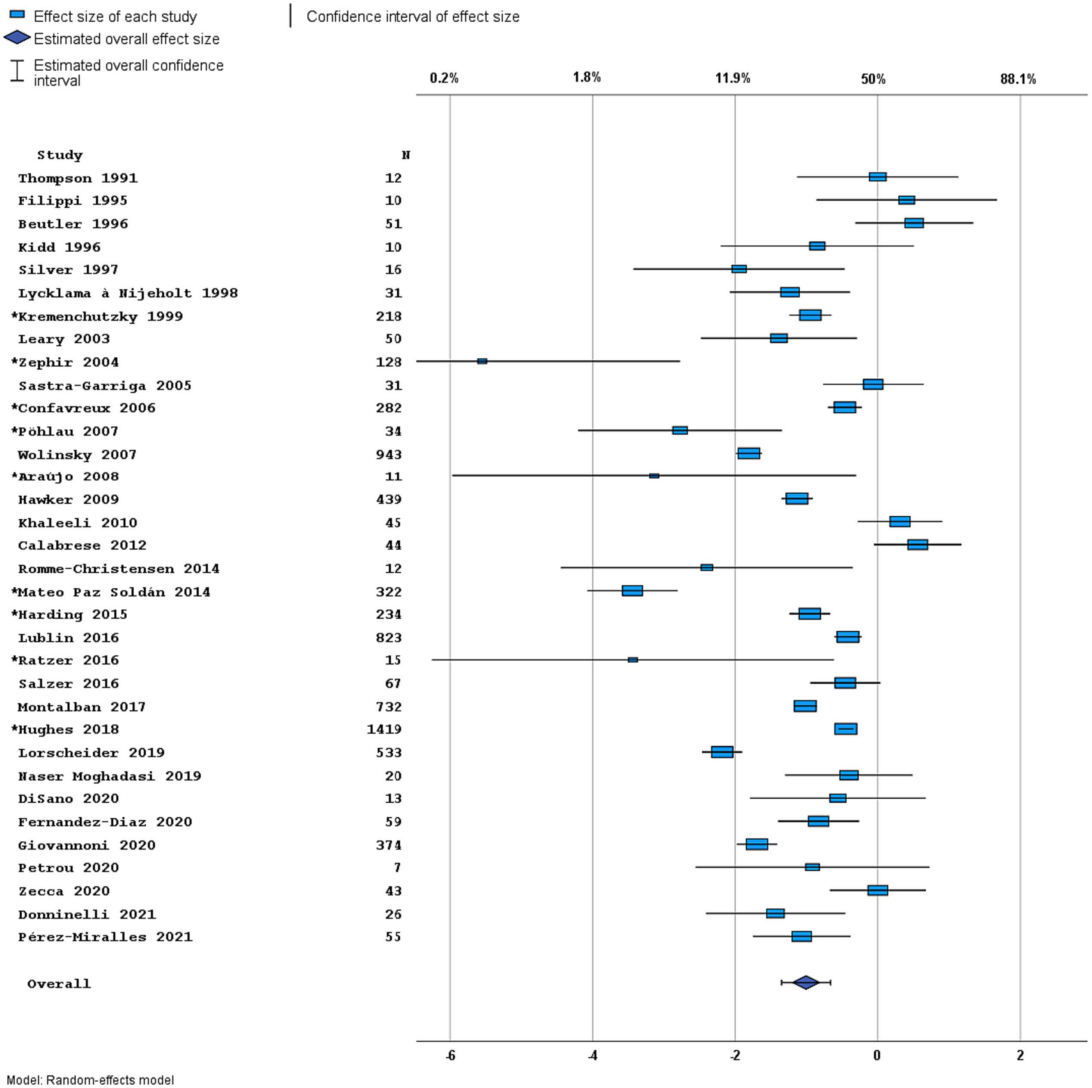
Forest plot of overall disease activity outcome. Random effects model. Lower axis shows logit scale, upper axis shows prevalence. *N*, number of total included people with PPMS. Studies with an asterisk (*) reported only data on clinical disease activity.

Most studies were performed in Europe. There was a wide variation in study design and inclusion criteria between the studies. The included studies are based on a total of 7,109 pwPPMS, with the number of pwPPMS per study ranging between 7 and 1,419, midpoint age at inclusion between 38.5–55.8 years, midpoint disease duration at inclusion between 3.0–11.7 years, and percentage of women between 0–73.3%. Twenty articles (5,008 pwPPMS) reported data on relapses, showing a weighted estimated proportion of pwPPMS with clinical disease activity of 9.2% (95% CI 5.3–15.6%) (Figure 2). Twenty-three articles (4,383 pwPPMS) reported data on radiological disease activity, showing a weighted estimated proportion of pwPPMS with radiological disease activity of 31.9% (95% CI 24.9–39.9%) (Figure 3). For overall disease activity in all studies – including studies with and without radiological outcome data – we found a weighted estimated proportion of pwPPMS with overall disease activity of 26.8% (95% CI 20.6–34.0%) (Figure 4). The heterogeneity of reported outcomes was very high (*I*^2^ 96.5% for relapses, 92.9% for GEL/NEL, 96.1% for overall disease activity). Meta-regression analyses were performed in an effort to explain some of this heterogeneity.

### 3.2. Meta-regression analyses

Results of both univariable and multivariable meta-regression analyses are summarized in Table 2.

**Table 2:** Results of meta-regression analysis.

Variable	Radiological disease activity			Clinical disease activity			Overall disease activity		
	*N*	*ß* estimate	*p*-value	*N*	*ß* estimate	*p*-value	*N*	*ß* estimate	*p*-value
Year of publication	23	−0.026	0.176*	20	−0.035	0.425	34	−0.015	0.454
Midpoint age at inclusion	23	−0.100	**0.017**	20	−0.057	0.384	34	−0.064	0.123*
Midpoint disease duration at inclusion (years)	23	−0.024	0.755	16	−0.103	0.591	30	−0.073	0.35
Midpoint EDSS at inclusion	23	−0.251	0.394	16	0.089	0.871	29	−0.433	0.093*
Percentage of female patients	18	0.004	0.853	19	0.016	0.569	29	−0.006	0.726
Percentage of patients on IMT	18	0.008	0.293	15	−0.013	0.249	26	−0.006	0.449
Follow-up time on study (years)	23	0.287	**0.008**	20	0.100	**0.017**	34	0.008	0.801
Dose of gadolinium used (mmol/kg)	11	2.107	0.331	–			–		
Number of MRI-scans	16	0.098	0.282	–			–		

Multivariable, including:	*N*	*ß* estimate	*p*-value	OR (95% CI)	*R* ^2^	*I* ^2^	*N*	*ß* estimate	*p*-value	OR (95% CI)	*R* ^2^	*I* ^2^	*N*	*ß* estimate	*p*-value	OR (95% CI)	*R* ^2^	*I* ^2^
Year of publication	*x*	−0.019	0.330	0.98 (0.95–1.02)														
Midpoint age at inclusion	*x*	−0.059	0.187	0.94 (0.87–1.03)			*x*	0.075	0.417	1.08 (0.91–1.28)			*x*	−0.093	**0.031**	0.91 (0.84–0.97)		
Midpoint disease duration at inclusion	*x*	−0.038	0.572	0.96 (0.856–1.10)			*x*	0.021	0.923	1.02 (0.67–1.56)			*x*	−0.058	0.432	0.94 (0.82–1.09)		
Midpoint EDSS at inclusion	*x*	−0.041	0.877	0.96 (0.58–1.59)			*x*	−0.459	0.470	0.63 (0.19–2.10)			*x*	−0.360	0.184	0.70 (0.42–1.17)		
Follow-up time on study	*x*	0.238	**0.033**	1.27 (1.04–1.55)			*x*	0.162	0.274	1.18 (0.89–1.55)			*x*	0.057	0.487	1.06 (0.27–4.23)		
	23				41.9	84.9	15				10.2	79.3	28				23.1	90.4

Random-effects model with continuity correction. *N*, number of included articles; EDSS, expanded disability status scale; IMT, immunomodulating therapy; OR, odds ratio; CI, confidence interval. *p < 0.2 in univariable analysis, therefore added to multivariable analysis.

#### 3.2.1. Clinical disease activity

Other than articles reporting no relapses, only four articles reported data on the annualized relapse rate (ARR) (30, 33, 41, 46), ranging between 0.04 and 0.15. In the other articles there were insufficient data to calculate an ARR. We therefore only performed analyses on percentage of patients with relapses, but not on ARR. In univariable analyses, follow-up duration showed a positive correlation with the prevalence of clinical disease activity in PPMS cohorts (*p* = 0.017): a longer follow-up predicted a higher prevalence of relapses. In multivariable analyses including the predefined variables (midpoint age, EDSS and disease duration-all at inclusion-and midpoint follow-up), none of the variables significantly predicted clinical disease activity. This multivariable model explained the variance in clinical disease activity for 10.2% (*R*^2^), and the unexplained heterogeneity remained substantial (*I*^2^ = 79.3%).

#### 3.2.2. Radiological disease activity

**Figure 5:**
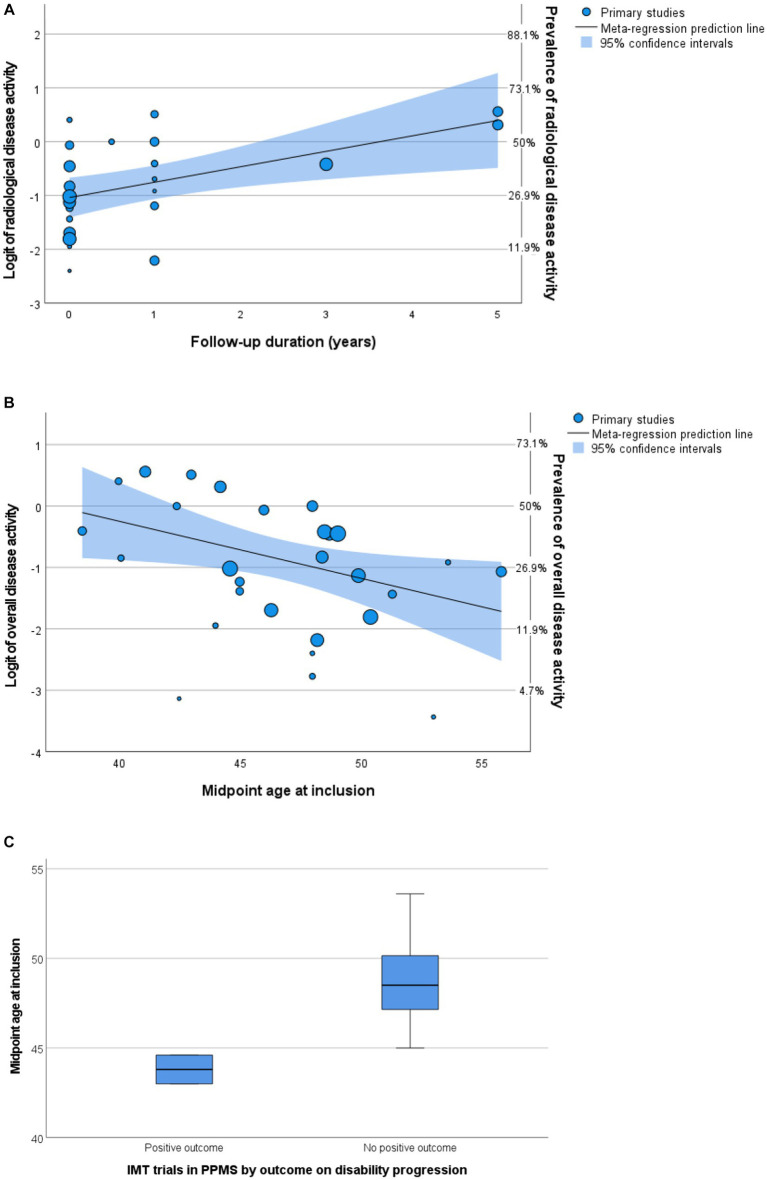
Predictors of disease activity. **(A,B)** Bubble plots of multivariable meta-regression analyses with continuity correction. Left Y-axis shows logit, right Y-axis shows prevalence. **(A)** Follow-up predicts radiological activity. **(B)** Midpoint age at inclusion predicts overall disease activity. **(C)** Boxplot of midpoint age at inclusion by outcome of randomized controlled trials. IMT, immunomodulating therapy; PPMS, primary progressive multiple sclerosis.

A total of seven articles included (cervical) spinal cord MRIs in their assessment of radiological disease activity (28, 32, 35, 50, 53–55), in three articles it was uncertain if MRI spinal cord was included (26, 27, 33). The number of MRIs made varied between 1 and 12. Most articles (*n* = 18) reported data on GEL, seven articles (also) reported data on new lesions, and no articles reported data on enlarging lesions. In univariable analyses, midpoint age at inclusion was a significant negative predictor for prevalence of radiological disease activity in PPMS cohorts (*p* = 0.017). The variable ‘radiological follow-up time’ was a significant positive predictor (*p* = 0.008), but this was not the case for ‘number of MRIs made’ (*p* = 0.282). In multivariable analyses including predefined variables and year of publication (*p* < 0.2 in univariable analysis), only radiological follow-up time remained a significant independent predictor [*p* = 0.033, OR 1.27 (95% CI 1.04–1.55), Figure 5A]. This multivariable model explained the variance in radiological disease activity for 41.9% (*R*^2^), but the unexplained heterogeneity remained high (*I*^2^ 84.9%).

#### 3.2.3. Overall disease activity

In univariable analyses, there were no significant predictors for overall disease activity. In multivariable analyses including predefined variables, only the midpoint age at inclusion was a significant negative predictor [*p* = 0.031, OR 0.91 (95% CI 0.84–0.99), Figure 5B]. This finding is in line with studies comparing active versus non active PPMS, reporting a younger age of onset in active PPMS (29, 30, 47). However, heterogeneity of the outcome remained high (*I*^2^ 90.4%) in this multivariable model and the variance in clinical disease activity was explained for only 23.1% (*R*^2^).

To explore the relevance of midpoint age for PPMS clinical trial outcomes, we compared the midpoint age at inclusion of randomized controlled trials in this meta-analysis with positive (5, 23) and negative (6, 28, 32, 34, 37, 38, 44) results on their disability progression endpoint. Indeed, positive trials showed a younger midpoint age at inclusion (median 43.8, range 43.0–44.6 years) compared to negative trials (median 48.5, range 45.0–53.6 years), although just missing the threshold of statistical significance (Mann–Whitney U test *p* = 0.056) (Figure 5C). Since inflammatory disease activity associates with an increased efficacy of IMTs, these findings support a higher prevalence of efficacy-associated inflammatory disease activity in younger participants.

#### 3.2.4. Sensitivity analysis

In the sensitivity analysis using a beta-binomial model, the conclusions with respect to the relevant predictors remained largely unchanged: a longer radiological follow-up time of the study predicted a higher prevalence of radiological disease activity (*p* = 0.005) and a lower midpoint age at inclusion predicted a higher prevalence of overall disease activity (*p* = 0.007). Also the effect sizes remained largely unchanged. Similar to the original analyses, no other statistically significant correlations were found.

### 3.3. Changes in PPMS cohort characteristics over time

**Figure 6:**
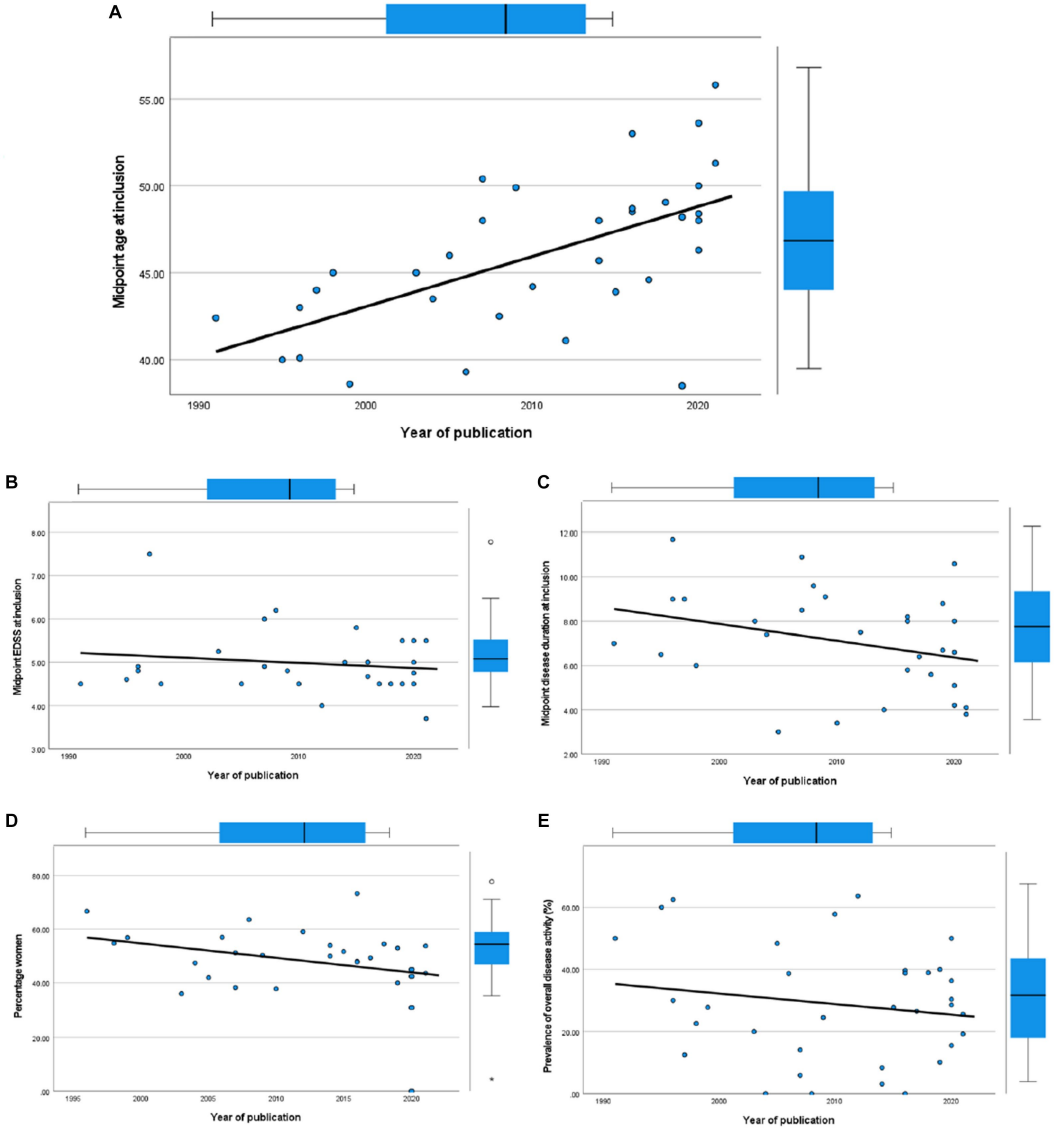
Scatterplots of cohort characteristics and year of publication, including corresponding boxplots on upper and right side. **(A)** Midpoint age at inclusion. **(B)** Midpoint EDSS at inclusion. **(C)** Midpoint disease duration at inclusion. **(D)** Percentage women. **(E)** Overall disease activity.

We investigated temporal changes in study populations of PPMS in terms of disease activity by performing correlation tests between year of publication and several cohort characteristics (Figure 6). We found a positive correlation with midpoint age at inclusion (Spearman’s *ρ* = 0.64, 95% CI 0.37–0.81). However, we found no significant correlation between year of publication and midpoint disease duration at inclusion (Spearman’s *ρ* = −0.332, 95% CI−0.625–0.043), midpoint EDSS at inclusion (Spearman’s *ρ* = −0.046, 95% CI−0.415–0.336), or between the percentage of women and year of publication (Spearman’s *ρ* = −0.310, 95% CI = −0.614–0.075). In line with the results of our meta-regression analyses, where year of publication did not predict disease activity, we found no significant correlation between year of publication and overall disease activity (Spearman’s *ρ* = −0.078, 95% CI−0.414–0.277).

## 4. Discussion

In this review we systematically analyzed the prevalence of several markers of inflammatory disease activity in pwPPMS as reported in literature. In addition, we investigated which cohort characteristics predicted disease activity. In summary, radiological disease activity was far more prevalent than clinical disease activity (31.9% versus 9.2%). Studies reporting only clinical outcomes pulled the weighted estimate of overall disease activity down to 26.8%. We found that midpoint age at inclusion was a negative predictor for overall disease activity. The follow-up duration was a positive predictor for radiological disease activity. The heterogeneity of reported disease activity outcomes remained high in our multivariable meta-regression analyses. This is likely due to the high variety of study designs we included, with different inclusion criteria, diagnostic criteria, and outcome measurements. Contrary to reported findings in RRMS (10, 11), we found no evidence for changes in disease activity in PPMS study populations in the last decades.

Our finding that a lower midpoint age at inclusion predicts a higher prevalence of disease activity, matches the finding of several included studies that active pwPPMS are younger than non-active pwPPMS (29, 30, 47). This finding is also in agreement with other studies that link age to different MS disease processes of inflammatory activity and disease progression (56–59). These results suggest that age is a major factor in inflammatory disease activity in MS. Although not statistically significant, the midpoint at inclusion in PPMS trials with positive effect of IMT (5, 23) seemed to be lower than in trials that did not show a positive effect (6, 28, 32, 34, 37, 38, 44). Therefore, age is an important factor to take into account when interpreting PPMS trial results. Our findings confirm that younger patients tend to have more inflammatory activity, and so younger study populations are likely to show more IMT efficacy (60). Likewise, Ziemssen et al. speculated on the possibility that variation in prevalence of disease activity in PPMS study cohorts is one of the drivers of variation in results of IMT trials in PPMS (61). Although they did not perform meta-analyses, they reported a large variation in the prevalence of disease activity including number of GEL and disease duration, possibly influencing the study outcomes. As debated by Portaccio et al. (4), this could indicate that, in pwPPMS with disease activity, a wide range of IMTs could be beneficial. This is especially important for regions in the world where ocrelizumab is unavailable or unaffordable.

We found that radiological activity was more prevalent than clinical activity (relapses) in PPMS cohorts, and was detected at a higher rate with a longer radiological follow-up-duration. Although we cannot extrapolate these findings from a cohort level directly to a patient level, it does seem to indicate that the odds of detecting disease activity in pwPPMS increase by performing longitudinal MRI scans over multiple years. As detection of disease activity can have consequences for the treatment of pwPPMS, our observations stress the need for prolonged active monitoring of inflammatory disease activity, especially in pwPPMS within the young age ranges. This follow-up requires a customized clinical and MRI-monitoring program dependent on patient characteristics and accessibility of diagnostics (62), and could putatively in the future be expanded by including biomarkers as NfL and GFAP (63–67). Although mostly studied in RRMS, these markers also were reported to have added value for prognostication and prediction of disease activity or progression in studies within progressive MS cohorts (68–70). Since these biomarkers are not yet widely integrated in clinical practice, we did not focus on these in our systematic review.

In correlation analyses between year of publication and cohort characteristics, we found that between 1991 and 2021, the age of pwPPMS at study inclusion has increased. This is similar to the finding by Nicholas et al. who studied changes in progressive MS trials over time (71). In contrast to Nicholas et al., we did not find an increase in midpoint disease duration or midpoint EDSS at inclusion despite this increasing age. Nor did we find evidence for a decrease in disease activity in PPMS study cohorts, contrary to reported findings in RRMS (10, 11). These findings could have several explanations. The changes in diagnostic criteria and phenotype definitions together with increasing therapeutic options for RRMS may have affected the neurologists’ appreciation of the distinction between PPMS and RRMS. Almost as a self-fulfilling prophecy, it could be that specifically the less active (and therefore likely older) people within the MS spectrum have been diagnosed with PPMS in the last decades (72). This does not explain our finding that there is no decrease of activity in PPMS study populations, but the latter might have been counterbalanced by increased radiological examinations. After the introduction of ocrelizumab as a treatment for PPMS in 2019, this incentive to diagnose any active MS patient as RRMS instead of PPMS might have been reduced. Because this treatment is so recent, we could not investigate differences in PPMS cohorts in this ocrelizumab era. A different explanation for the increased age at inclusion of PPMS cohorts might be that due to overall improvement in health and environmental factors such as decreased tobacco smoking (10, 13), symptoms or even pathology of (PP)MS start at a later age and progress more slowly.

### 4.1. Strengths and limitations

Some limitations of our review need to be addressed. Firstly, we investigated possible predictive properties of cohort characteristics on disease activity prevalence. This means we cannot apply these predictors on a patient level. In addition, we do not have the data to better understand these properties on a biological level. However, some of our findings have previously been reported to be predictive of disease activity on a patient level. Our findings do provide additional evidence for these conclusions. Secondly, we have chosen to include a wide variety of studies, both in study design and in methodological quality. This contributed to a high heterogeneity of reported outcomes, which in turn was difficult to explain with our cohort characteristics. On the other hand, it is because of this wide variety of the included studies that we believe we have given a thorough overview of disease activity in PPMS cohorts. Our results can help to interpret PPMS study results, especially concerning therapeutic trials.

## 5. Conclusion

The pooled estimate of inflammatory disease activity in PPMS is 26.8%, indicating that about a quarter of pwPPMS could benefit meaningfully from IMT. In contrast to RRMS, there are no significant changes in prevalence of disease activity in PPMS cohorts over time. Midpoint age at inclusion is the most important predictive factor for disease activity in PPMS cohorts, and a longer follow-up duration associates with a higher prevalence of detecting radiological disease activity in PPMS. Therefore, we advise that, especially when treating relatively young pwPPMS, clinicians should remain vigilant for inflammatory disease activity and should follow-up pwPPMS accordingly. This follow-up should include a customized program of clinical and MRI monitoring to not overlook possible indications for IMT. In addition, the age of included patients should be carefully considered when interpreting PPMS trial results.

## Data availability statement

The original contributions presented in the study are included in the article/Supplementary material, further inquiries can be directed to the corresponding authors.

## Author contributions

KB: Conceptualization, Data curation, Formal analysis, Investigation, Methodology, Project administration, Visualization, Writing – original draft. JR: Methodology, Supervision, Writing – review & editing. NT: Data curation, Investigation, Writing – review & editing. JS: Conceptualization, Methodology, Supervision, Writing – review & editing. BW: Conceptualization, Data curation, Methodology, Supervision, Writing – review & editing. JB: Conceptualization, Data curation, Investigation, Methodology, Supervision, Writing – review & editing.
