# Supplementary material for: Disease activity in primary progressive multiple sclerosis: a systematic review and meta-analysis
Source: Front Neurol. 2023 Nov 6;14:1277477. doi: 10.3389/fneur.2023.1277477 (PMC10661414; doi:10.3389/fneur.2023.1277477)
Supplement: Supplementary file 2 [file Data_Sheet_2.docx]

**S2: Search terms**

**embase.com (1971-)**

('multiple sclerosis'/de OR (MS OR (multiple NEAR/3 sclerosis)):ab,ti) AND ((primary NEAR/3 progressiv*) OR ppms OR ((progressive NEAR/3 (ms OR multiple-sclerosis)) NOT ((secondary) NOT (primary)))):ab,ti,kw AND ('inflammation'/exp OR 'antiinflammatory activity'/de OR 'autoimmunity'/de OR 'autoimmune disease'/de OR 'antiinflammatory agent'/de OR (inflammat* OR antiinflammat* OR autoimmun* OR auto-immun*):ab,ti,kw) NOT ([animals]/lim NOT [humans]/lim)

**Medline ALL Ovid (1946-)**

(Multiple Sclerosis/ OR Multiple Sclerosis, Chronic Progressive/ OR (MS OR (multiple ADJ3 sclerosis)).ab,ti.) AND ((primary ADJ3 progressiv*) OR ppms OR ((progressive ADJ3 (ms OR multiple-sclerosis)) NOT ((secondary) NOT (primary)))).ab,ti,kw. AND (exp Inflammation/ OR Autoimmunity/ OR Autoimmune Diseases/ OR Anti-Inflammatory Agents/ OR (inflammat* OR antiinflammat* OR autoimmun* OR auto-immun*).ab,ti,kw.) NOT (exp animals/ NOT humans/)

**Web of science Core Collection (1992-)**

TS=(((MS OR (multiple NEAR/2 sclerosis))) AND ((primary NEAR/2 progressiv*) OR ppms OR ((progressive NEAR/2 (ms OR multiple-sclerosis)) NOT ((secondary) NOT (primary)))) AND ((inflammat* OR antiinflammat* OR autoimmun* OR auto-immun*)))

**Cochrane CENTRAL register of trials (1992-)**

((MS OR (multiple NEAR/3 sclerosis)):ab,ti) AND ((primary NEAR/3 progressiv*) OR ppms OR ((progressive NEAR/3 (ms OR multiple-sclerosis)) NOT ((secondary) NOT (primary)))):ab,ti,kw AND ((inflammat* OR antiinflammat* OR autoimmun* OR auto-immun*):ab,ti,kw)

**Google Scholar**

"primary progressive ms"|"primary progressive multiple sclerosis" inflammation|inflammatory|antiinflammation|antiinflammatory|autoimmunity|autoimmune|"auto immunity|immune"

**Search on March 30^th^, 2020**

| **Database** | **Number of hits** | **Number of hits after duplicate removal** |
| --- | --- | --- |
| embase.com (1971-) | 2355 | 2291 |
| Medline ALL Ovid (1946-) | 1042 | 146 |
| Web of science Core Collection (1992-) | 1525 | 572 |
| Cochrane CENTRAL register of trials (1992-) | 135 | 40 |
| Google Scholar | 200 | 101 |
| **Total** | **5257** | **3150** |

**Search on August 27^th^, 2020**

| **Database** | **Number of hits** | **Number of hits after duplicate removal** |
| --- | --- | --- |
| embase.com (1971-) | 2518 | 2438 |
| Medline ALL Ovid (1946-) | 1087 | 138 |
| Web of science Core Collection (1992-) | 1572 | 572 |
| Cochrane CENTRAL register of trials (1992-) | 144 | 39 |
| Google Scholar (200 top-ranked) | 200 | 100 |
| **Total** | **5521** | **3287** |

*New references: 209*

**Search on June 7^th^ 2021**

| **Database** | **Number of hits** | **Number of hits after duplicate removal** |
| --- | --- | --- |
| embase.com (1971-) | 2734 | 2623 |
| Medline ALL Ovid (1946-) | 1173 | 141 |
| Web of science Core Collection (1992-) | 1669 | 583 |
| Cochrane CENTRAL register of trials (1992-) | 159 | 46 |
| Google Scholar (200 top-ranked) | 200 | 109 |
| **Total** | **5935** | **3502** |

*New references: 290*
