# Supplementary material for: Disease activity in primary progressive multiple sclerosis: a systematic review and meta-analysis
Source: Front Neurol. 2023 Nov 6;14:1277477. doi: 10.3389/fneur.2023.1277477 (PMC10661414; doi:10.3389/fneur.2023.1277477)
Supplement: Supplementary file 3 [file Data_Sheet_3.docx]

**S3: Reasons for exclusion in full text assessment**

| **First Author** | **Year of publication** | **Full Text NL/ENG?** | **Original research?** | **Five or more 5 PPMS patients?** | **Sufficient data on PPMS cohort?** | **Data on REL, GEL and/or NEL?** | **Unique cohort for this review?** | **Inclusion?** | **Notes** |
| --- | --- | --- | --- | --- | --- | --- | --- | --- | --- |
| **Alping** | 2016 | No |  |  |  |  |  | No | Conference abstract. |
| **Alvarez** | 2015 | No |  |  |  |  |  | No | Poster |
| **Androdias** | 2008 | No |  |  |  |  |  | No | Citation |
| **Araujo** | 2008 | Yes | Yes | Yes | Yes | Yes | Yes | Yes |  |
| **Bar-or** | 2020 | No |  |  |  |  |  | No | Conference abstract |
| **Bellmann-Strobl** | 2006 | No |  |  |  |  |  | No | No full text available |
| **Bensi** | 2019 | No |  |  |  |  |  | No | Conference abstract |
| **Bergman** | 2021 | Yes | Yes | Yes | No |  |  | No | No age data of PPMS patients |
| **Bermel** | 2010 | Yes | Yes | Yes | No |  |  | No | No age data of PPMS patients |
| **Bernal Varela** | 2020 | No |  |  |  |  |  | No | Poster |
| **Beutler** | 1996 | Yes | Yes | Yes | Yes | Yes | Yes | Yes |  |
| **Birnbaum** | 2014 | No |  |  |  |  |  | No | Conference abstract |
| **Blum** | 2010 | No |  |  |  |  |  | No | Poster |
| **Boffa** | 2020 | No |  |  |  |  |  | No | Poster |
| **Bolling** | 2020 | No |  |  |  |  |  | No | Poster |
| **Bonab** | 2012 | Yes | Yes | No |  |  |  | No | Not enough PPMS patients |
| **Bowen** | 2012 | Yes | Yes | Yes | Yes | No |  | No | No data on disease activity in PPMS patients |
| **Bowen** | 2003 | Yes | Yes | Yes | No |  |  | No | No age data f PPMS patients |
| **Brady** | 2020 | No |  |  |  |  |  | No | Poster |
| **Bramow** | 2020 | No |  |  |  |  |  | No | Poster |
| **Brola** | 2019 | Yes | Yes | Yes | Yes | No |  | No | No data on disease activity in PPMS patients |
| **Buttmann** | 2020 | No |  |  |  |  |  | No | Poster |
| **Cagol** | 2020 | No |  |  |  |  |  | No | Poster |
| **Calabrese** | 2012 | Yes | Yes | Yes | Yes | Yes | Yes | Yes |  |
| **Calabrese** | 2009 | Yes | Yes | Yes | Yes | Yes | No | No | Same cohort as Calabrese 2012 |
| **Castillo-Trivino** | 2013 | Yes | No |  |  |  |  | No | Systematic review |
| **Cellerino** | 2020 | No |  |  |  |  |  | No | Poster |
| **Choi** | 2019 | No |  |  |  |  |  | No | Conference Abstract |
| **Ciampi** | 2019 | No |  |  |  |  |  | No | Conference abstract |
| **Ciampi** | 2020 | Yes | Yes | Yes | No |  |  | No | No age data of PPMS patients |
| **Coban** | 2020 | No |  |  |  |  |  | No | Poster |
| **Cook** | 1995 | Yes | Yes | No |  |  |  | No | Unclear if cohort contains SPMS and/or PPMS |
| **Correale** | 2000 | Yes | Yes | No |  |  |  | No | SPMS |
| **Cottrell** | 1999 | Yes | Yes | Yes | Yes | No |  | No | No data on disease activity in PPMS patients |
| **Couloume** | 2020 | Yes | Yes | Yes | No |  |  | No | No age data of PPMS patients, no definition of PPMS |
| **Cubas-Nunez** | 2021 | Yes | Yes | Yes | Yes | No |  | No | Data on disease activity only in combined PPMS/SPMS group |
| **Cull** | 2017 | Yes | Yes | No |  |  |  | No | Not enough PPMS patients |
| **Debouverie** | 2004 | Yes | Yes | Yes | No |  |  | No | No age data of PPMS patients |
| **Dehmeshki** | 2003 | Yes | Yes | Yes | Yes | No |  | No | No data on disease activity in PPMS patients |
| **DiSano** | 2020 | Yes | Yes | Yes | Yes | Yes | Yes | Yes |  |
| **Donninelli** | 2021 | Yes | Yes | Yes | Yes | Yes | Yes | Yes |  |
| **Elliiot** | 2019 | Yes | Yes | Yes | Yes | Yes | No | No | ORATORIO cohort, already included in Montalban 2017 |
| **Elliot** | 2017 | No |  |  |  |  |  | No | Poster |
| **Emersic** | 2020 | No |  |  |  |  |  | No | Poster |
| **Epstein** | 2020 | No |  |  |  |  |  | No | Poster |
| **Fainardi** | 2002 | Yes | Yes | Yes | No |  |  | No | No age data of PPMS patients |
| **Fakih** | 2018 | Yes | Yes | Yes | No |  |  | No | No age data of PPMS patients |
| **Fan** | 2011 | No |  |  |  |  |  | No | Chinese language |
| **Fassas** | 2002 | Yes | Yes | Yes | No |  |  | No | No age data of PPMS patients, no definition of PPMS |
| **Fernandez-Diaz** | 2021 | Yes | Yes | Yes | Yes | Yes | Yes | Yes |  |
| **Fernandez-Velasco** | 2020 | No |  |  |  |  |  | No | Poster |
| **Filippi** | 2020 | Yes | No |  |  |  |  | No | Review |
| **Filippi** | 1995 | Yes | Yes | Yes | Yes | Yes | Yes | Yes |  |
| **Filippi** | 2000 | Yes | Yes | Yes | No |  |  | No | No age data of PPMS patients, no definition of PPMS |
| **Fissolo** | 2021 | Yes | Yes | Yes | Yes | No |  | No | Unfit outcome measure of disease activity |
| **Gascon** | 2014 | No |  |  |  |  |  | No | Poster |
| **Giarraputo** | 2020 | No |  |  |  |  |  | No | Poster |
| **Giovannoni** | 2020 | Yes | Yes | Yes | Yes | Yes | Yes | Yes |  |
| **Groen** | 2021 | No |  |  |  |  |  | No | Editorial |
| **Guerrieri** | 2020 | No |  |  |  |  |  | No | ‘Platform presentations’ |
| **Haghikia** | 2017 | Yes | No |  |  |  |  | No | Editorial commentary |
| **Harding** | 2015 | Yes | Yes | Yes | Yes | Yes | Yes | Yes |  |
| **Harding** | 2012 | No |  |  |  |  |  | No | Poster |
| **Hartung** | 2002 | Yes | Yes | Yes | No |  |  | No | No age data of PPMS patients |
| **Hawker** | 2010 | Yes | No |  |  |  |  | No | Reports from consensus meeting |
| **Hawker** | 2009 | Yes | Yes | Yes | Yes | Yes | Yes | Yes |  |
| **Hellwig** | 2006 | Yes | Yes | Yes | No |  |  | No | No age data of PPMS patients, no definition of PPMS |
| **Hildesheim** | 2020 | No |  |  |  |  |  | No | Poster |
| **Hoffmann** | 2006 | Yes | Yes | Yes | No |  |  | No | No age data of PPMS patients, no definition of PPMS |
| **Hohol** | 1999 | Yes | Yes | No |  |  |  | No | Unclear if cohort contains SPMS and/or PPMS |
| **Holm** | 2015 | No |  |  |  |  |  | No | Poster |
| **Hommes** | 1975 | Yes | Yes | Yes | Yes | No |  | No | No data on disease activity in PPMS patients |
| **Hughes** | 2018 | Yes | Yes | Yes | Yes | Yes | Yes | Yes | MSBase registry |
| **Hughes** | 2017 | No |  |  |  |  |  | No | Poster |
| **Iacobaeus** | 2019 | Yes | Yes | No |  |  |  | No | Not enough PPMS patients |
| **Ingle** | 2005 | Yes | Yes | Yes | Yes | Yes | No | No | Same cohort as Khaleeli 2010 |
| **Jelcic** | 2020 | No |  |  |  |  |  | No | Poster |
| **Kamma** | 2020 | No |  |  |  |  |  | No | Poster |
| **Karussis** | 2012 | Yes | Yes | No |  |  |  | No | Only 'relapsing progressive' |
| **Khaleeli** | 2008 | Yes | Yes | Yes | Yes | No |  | No | No data on disease activity in PPMS patients |
| **Khaleeli** | 2007 | Yes | Yes | Yes | Yes | No |  | No | No data on disease activity in PPMS patients |
| **Khaleeli** | 2010 | Yes | Yes | Yes | Yes | Yes | Yes | Yes |  |
| **Kharya** | 2019 | No |  |  |  |  |  | No | Poster |
| **Khatri** | 1988 | No |  |  |  |  |  | No | No full text available |
| **Kirgizov** | 2019 | No |  |  |  |  |  | No | Poster |
| **Kis** | 2008 | Yes | Yes | Yes | No |  |  | No | No age data of PPMS patients |
| **Kleiter** | 2009 | No |  |  |  |  |  | No | Poster |
| **Koch** | 2019 | No |  |  |  |  |  | No | Poster |
| **Komori** | 2016 | Yes | Yes | No |  |  |  | No | Only SPMS patients |
| **Leary** | 2003 | Yes | Yes | Yes | Yes | Yes | Yes | Yes |  |
| **Leonidou** | 2019 | Yes | Yes | No |  |  |  | No | Not enough PPMS patients |
| **Liegey** | 2018 | No |  |  |  |  |  | No | Poster |
| **Lorscheider** | 2017 | No |  |  |  |  |  | No | Poster |
| **Lorscheider** | 2019 | Yes | Yes | Yes | Yes | Yes | Yes | Yes |  |
| **Losseff** | 1996 | Yes | Yes | Yes | Yes | Yes | No | No | Same cohort as Thompson 1991 |
| **Lublin** | 2016 | Yes | Yes | Yes | Yes | Yes | Yes | Yes |  |
| **Luckey** | 2012 | No |  |  |  |  |  | No | Poster |
| **Luna Higuera** | 2021 | No |  |  |  |  |  | No | Poster |
| **Maciejowski** | 2015 | No |  |  |  |  |  | No | Polish language |
| **Maljaei** | 2020 | No |  |  |  |  |  | No | Poster |
| **Malucchi** | 2017 | No |  |  |  |  |  | No | Poster |
| **Marastoni** | 2020 | No |  |  |  |  |  | No | Poster |
| **Marrodan** | 2019 | No |  |  |  |  |  | No | Poster |
| **Mathais** | 2020 | Yes | Yes | Yes | No |  |  | No | No age data of PPMS patients, no definition of PPMS |
| **Mayer** | 2019 | Yes | Yes | Yes | Yes | Yes | No | No | ORATORIO cohort, already included in Montalban 2017 |
| **McDonnell** | 1998 | Yes | Yes | Yes | Yes | No |  | No | No data on disease activity in PPMS patients |
| **Midaglia** | 2020 | No |  |  |  |  |  | No | Poster |
| **Miller** | 2004 | Yes | Yes | Yes | Yes | Yes | No | No | Interferon trial cohort, already included in Leary 2003 |
| **Miller** | 2018 | Yes | Yes | Yes | Yes | Yes | No | No | INFORMS cohort, already included in Lublin 2016 |
| **Mohammadi** | 2021 | No |  |  |  |  |  | No | “withdrawn upon the request of the author due to the errors and invalid information in the article” |
| **Montalban** | 2017 | Yes | Yes | Yes | Yes | Yes | Yes | Yes |  |
| **Montalban** | 2009 | Yes | Yes | Yes | No |  |  | No | No age data of PPMS patients (tMS and PPMS grouped together) |
| **Morrison** | 2020 | No |  |  |  |  |  | No | Poster |
| **Moviglia** | 2013 | No |  |  |  |  |  | No | Conference abstract |
| **Mueller** | 2020 | No |  |  |  |  |  | No | Poster |
| **Muller** | 2014 | Yes | Yes | Yes | No |  |  | No | No age data of PPMS patients, no definition of PPMS |
| **Muller** | 2016 | Yes | Yes | Yes | No |  |  | No | No age data of PPMS patients, no definition of PPMS |
| **Munoz-San Martin** | 2019 | No |  |  |  |  |  | No | Poster |
| **Murrietta-Alvarez** | 2021 | Yes | Yes | Yes | No |  |  | No | No age data of PPMS patients, no definition of PPMS |
| **Nabavi** | 2013 | No |  |  |  |  |  | No | Conference abstract |
| **Naegelin** | 2020 | Yes | Yes | Yes | Yes | No |  | No | No data on disease activity in PPMS patients |
| **Naismith** | 2021 | Yes | Yes | Yes | No |  |  | No | No age data of PPMS patients, no definition of PPMS |
| **Naser Moghadasi** | 2019 | Yes | Yes | Yes | Yes | Yes | Yes | Yes |  |
| **Nayak** | 2020 | Yes | Yes | No |  |  |  | No | No separate PPMS group defined |
| **Ni** | 2006 | Yes | Yes | No |  |  |  | No | Not enough PPMS patients |
| **Novik (BMT)** | 2011 | No |  |  |  |  |  | No | Poster |
| **Novik (MS)** | 2011 | No |  |  |  |  |  | No | Poster |
| **Nylund** | 2019 | No |  |  |  |  |  | No | Poster |
| **Perez-Miralles** | 2021 | Yes | Yes | Yes | Yes | Yes | Yes | Yes |  |
| **Petrou** | 2020 | Yes | Yes | Yes | Yes | Yes | Yes | Yes | Data on characteristics of PPMS patients in supplementary materials |
| **Pohlau** | 2007 | Yes | Yes | Yes | Yes | Yes | Yes | Yes |  |
| **Ratzer** | 2016 | Yes | Yes | Yes | Yes | Yes | Yes | Yes |  |
| **Ratzer** | 2013 | No |  |  |  |  |  | No | Poster |
| **Rhone** | 2018 | No |  |  |  |  |  | No | Poster |
| **Rice** | 2000 | Yes | Yes | Yes | No |  |  | No | No age data of PPMS patients |
| **Robles** | 2017 | No |  |  |  |  |  | No | Poster |
| **Romme Christensen** | 2012 | No |  |  |  |  |  | No | Poster |
| **Romme Christensen** | 2016 | No |  |  |  |  |  | No | Poster |
| **Romme-Christensen** | 2014 | Yes | Yes | Yes | Yes | Yes | Yes | Yes |  |
| **Ruggieri** | 2018 | No |  |  |  |  |  | No | Poster |
| **Sadiq** | 2010 | Yes | Yes | Yes | No |  |  | No | Insufficient data about characteristics PPMS patients |
| **Safi** | 2020 | No |  |  |  |  |  | No | Poster |
| **Sajja** | 2008 | Yes | Yes | Yes | Yes | Yes | No | No | PROMiSE cohort, already included in Wolinsky 2007 |
| **Salzer** | 2016 | Yes | Yes | Yes | Yes | Yes | Yes | Yes | Swedish MS registry |
| **Sastre-Garriga** | 2005 | Yes | Yes | Yes | Yes | Yes | Yes | Yes |  |
| **Sastre-Garriga** | 2003 | No |  |  |  |  |  | No | Citation |
| **Scalfari** | 2015 | No |  |  |  |  |  | No | Poster |
| **Schiavi** | 2021 | Yes | Yes | No |  |  |  | No | Unclear if cohort contains SPMS and/or PPMS |
| **Seibert** | 2015 | No |  |  |  |  |  | No | Conference abstract |
| **Sellebjerg** | 2017 | Yes | Yes | Yes | Yes | Yes | No | No | Combination from two already included cohorts from Ratzer 2016 and Romme Christensen 2014 |
| **Singh** | 2020 | No |  |  |  |  |  | No | Poster |
| **Sipe** | 1994 | Yes | Yes | No |  |  |  | No | Unclear if cohort contains SPMS and/or PPMS |
| **Smoot** | 2020 | No |  |  |  |  |  | No | Poster |
| **Smoot** | 2019 | No |  |  |  |  |  | No | Poster |
| **Soldan** | 2013 | No |  |  |  |  |  | No | Conference abstract |
| **Stark** | 2016 | No |  |  |  |  |  | No | Conference abstract |
| **Tagge** | 2020 | No |  |  |  |  |  | No | Poster |
| **Thompson** | 1991 | Yes | Yes | Yes | Yes | Yes | Yes | Yes |  |
| **Tumani** | 2012 | No |  |  |  |  |  | No | Conference abstract |
| **Tur** | 2011 | Yes | Yes | Yes | No |  |  | No | No age data of PPMS patients (tMS and PPMS together, same cohort as Montalban 2009) |
| **Tur** | 2016 | No |  |  |  |  |  | No | Poster |
| **Uccelli** | 2020 | No |  |  |  |  |  | No | Conference abstract |
| **Vermersch** | 2012 | Yes | Yes | Yes | No |  |  | No | No age data of PPMS patients |
| **Wang** | 2018 | Yes | Yes | Yes | No |  |  | No | No age data of PPMS patients, unfit outcome measures of disease activity |
| **Wolinksy** | 2018 | Yes | Yes | Yes | Yes | Yes | No | No | PROMiSE cohort, already included in Wolinsky 2007 |
| **Wolinksy (MS)** | 2016 | No |  |  |  |  |  | No | Poster |
| **Wolinsky** | 2017 | Yes | Yes | Yes | Yes | Yes | No | No | ORATORIO cohort, already included in Montalban 2017 |
| **Wolinsky** | 2007 | Yes | Yes | Yes | Yes | Yes | Yes | Yes |  |
| **Wolinsky** | 2020 | Yes | Yes | Yes | Yes | Yes | No | No | ORATORIO cohort, already included in Montalban 2017 |
| **Wolinsky (MS)** | 2016 | No |  |  |  |  |  | No | Poster |
| **Zecca** | 2020 | Yes | Yes | Yes | Yes | Yes | Yes | Yes |  |
| **Zephir** | 2004 | Yes | Yes | Yes | Yes | Yes | Yes | Yes |  |
| **Zephir** | 2005 | Yes | Yes | Yes | Yes | Yes | No | No | Cohort already included in Zephir 2004 |
| **Zephir** | 2002 | No |  |  |  |  |  | No | French |
| **Ziliotto** | 2020 | Yes | Yes | No |  |  |  | No | Unclear if cohort contains SPMS and/or PPMS |
| **Additional inclusions via references** | | | | | | | | | |
| **Confavreux** | 2006 | Yes | Yes | Yes | Yes | Yes | Yes | Yes |  |
| **Lycklama a Nijeholt** | 1998 | Yes | Yes | Yes | Yes | Yes | Yes | Yes |  |
| **Mateo Paz Soldan** | 2014 | Yes | Yes | Yes | Yes | Yes | Yes | Yes |  |
| **Kidd** | 1996 | Yes | Yes | Yes | Yes | Yes | Yes | Yes |  |
| **Kremenchutzky** | 1999 | Yes | Yes | Yes | Yes | Yes | Yes | Yes |  |
| **Siver** | 1997 | Yes | Yes | Yes | Yes | Yes | Yes | Yes |  |
| S2: reason for exclusion based on full text assessment. Abbreviations: NL= Nederlands (Dutch language), ENG= English, PPMS= primary progressive multiple sclerosis, REL= relapses, GEL= gadolinium enhancing lesions, NEL= new or clearly enlarging lesions, tMS= transitional multiple sclerosis, SPMS= secondary progressive multiple sclerosis | | | | | | | | | |
