# Supplementary material for: Disease activity in primary progressive multiple sclerosis: a systematic review and meta-analysis
Source: Front Neurol. 2023 Nov 6;14:1277477. doi: 10.3389/fneur.2023.1277477 (PMC10661414; doi:10.3389/fneur.2023.1277477)
Supplement: Supplementary file 4 [file Data_Sheet_4.docx]

| **Study** | **Study design** | **Methodological quality criteria** | **Responses** | | | |
| --- | --- | --- | --- | --- | --- | --- |
|  |  |  | **Yes** | **No** | **Can't tell** | **Comments** |
| **Araujo** | **Screening questions (all types)** | S1. Are there clear research questions? | Yes |  |  | Research question is described, but vague: 'evaluate the use of IVMP in PPMS'. |
|  |  | S2. Do the collected data allow to address the research questions? |  |  | Can’t tell | No control group. |
|  | **4. Quantitative descriptive:**  **Case-series** | 4.1. Is the sampling strategy relevant to address the research question? |  | No |  | Unclear how the patient group was formed. |
|  |  | 4.2. Is the sample representative of the target population? |  | No |  | Included patients were very young for PPMS, a lot of optic neuritis, unsure which McDonald criteria were used. |
|  |  | 4.3. Are the measurements appropriatie? |  |  | Can’t tell | No standardized follow-up protocol. |
|  |  | 4.4. Is the risk of nonresponse bias low? |  |  | Can’t tell | Unclear representation of data. |
|  |  | 4.5. Is the statistical analysis appropriate to answer the research question? |  |  | Can’t tell | No statistical analyses performed. |
|  | **Total** | Score: | **0/5** |  |  |  |
| **Beutler** | **Screening questions (all types)** | S1. Are there clear research questions? | Yes |  |  |  |
|  |  | S2. Do the collected data allow to address the research questions? | Yes |  |  |  |
|  | **2. Quantitative randomized controlled trials** | 2.1. Is randomization appropriately performed? | Yes |  |  |  |
|  |  | 2.2 Are the groups comparable at baseline? | Yes |  |  |  |
|  |  | 2.3 Are there complete outcome data? | Yes |  |  | Data collection and MRI protocol were well described. |
|  |  | 2.4. Are outcome assessors blinded to the intervention provided? | Yes |  |  |  |
|  |  | 2.5. Did the participants adhere to the assigned intervention? |  | No |  | Multiple deviations from protocol were described. |
|  | **Total** | Score: | **4/5** |  |  |  |
| **Calabrese** | **Screening questions (all types)** | S1. Are there clear research questions? | Yes |  |  |  |
|  |  | S2. Do the collected data allow to address the research questions? | Yes |  |  |  |
|  | **3. Quantitative non-randomized:**  **Cohort** | 3.1. Are all the participants representative to the target population? | Yes |  |  |  |
|  |  | 3.2. Are the measurements appropriate regarding both the outcome and intervention (or exposure?) | Yes |  |  |  |
|  |  | 3.3. Are there complete outcome data? | Yes |  |  |  |
|  |  | 3.4. Are the confounders accounted for in the design and analysis? | Yes |  |  |  |
|  |  | 3.5. During the study period, is the intervention administered (or exposure occurred) as intended? | Yes |  |  |  |
|  | **Total** | Score: | **5/5** |  |  |  |
| **Confavreux** | **Screening questions (all types)** | S1. Are there clear research questions? | Yes |  |  |  |
|  |  | S2. Do the collected data allow to address the research questions? | Yes |  |  |  |
|  | **3. Quantitative non-randomized:**  **Real-world, registry.** | 3.1. Are all the participants representative to the target population? | Yes |  |  |  |
|  |  | 3.2. Are the measurements appropriate regarding both the outcome and intervention (or exposure?) |  | No |  | There was no standardized follow-up, datacollection was partly retrospective. |
|  |  | 3.3. Are there complete outcome data? | Yes |  |  |  |
|  |  | 3.4. Are the confounders accounted for in the design and analysis? | Yes |  |  |  |
|  |  | 3.5. During the study period, is the intervention administered (or exposure occurred) as intended? | Yes |  |  |  |
|  | **Total** | Score: | **4/5** |  |  |  |
| **Disano** | **Screening questions (all types)** | S1. Are there clear research questions? | Yes |  |  |  |
|  |  | S2. Do the collected data allow to address the research questions? | Yes |  |  |  |
|  | **3. Quantitative non-randomized:**  **Cohort** | 3.1. Are all the participants representative to the target population? | Yes |  |  |  |
|  |  | 3.2. Are the measurements appropriate regarding both the outcome and intervention (or exposure?) |  |  | Can’t tell | Multiple outcome measures were taken together. There was so clear radiological or clinical follow-up protocol described. |
|  |  | 3.3. Are there complete outcome data? | Yes |  |  |  |
|  |  | 3.4. Are the confounders accounted for in the design and analysis? |  | No |  | Possible confounding between: disease activity – treatment – biomarker. |
|  |  | 3.5. During the study period, is the intervention administered (or exposure occurred) as intended? | Yes |  |  |  |
|  | **Total** | Score: | **3/5** |  |  |  |
| **Donnineli** | **Screening questions (all types)** | S1. Are there clear research questions? | Yes |  |  |  |
|  |  | S2. Do the collected data allow to address the research questions? | Yes |  |  |  |
|  | **3. Quantative non-randomized**  **Cohort** | 3.1. Are all the participants representative to the target population? | Yes |  |  |  |
|  |  | 3.2. Are the measurements appropriate regarding both the outcome and intervention (or exposure?) | Yes |  |  |  |
|  |  | 3.3. Are there complete outcome data? | Yes |  |  |  |
|  |  | 3.4. Are the confounders accounted for in the design and analysis? | Yes |  |  |  |
|  |  | 3.5. During the study period, is the intervention administered (or exposure occurred) as intended? | Yes |  |  |  |
|  | **Total** | Score: | **5/5** |  |  |  |
| **Fernandez-Diaz** | **Screening questions (all types)** | S1. Are there clear research questions? | Yes |  |  |  |
|  |  | S2. Do the collected data allow to address the research questions? | Yes |  |  | For safety question: yes. For effectivity question: no (no control group). |
|  | **3. Quantative non-randomized**  **Before-and-after study** | 3.1. Are all the participants representative to the target population? | Yes |  |  |  |
|  |  | 3.2. Are the measurements appropriate regarding both the outcome and intervention (or exposure?) |  | No |  | The measurements of MRI and clinical outcomes were only partly standardized. |
|  |  | 3.3. Are there complete outcome data? | Yes |  |  |  |
|  |  | 3.4. Are the confounders accounted for in the design and analysis? |  | No |  | No control; not adjusted for possible placebo effect. |
|  |  | 3.5. During the study period, is the intervention administered (or exposure occurred) as intended? | Yes |  |  |  |
|  | **Total** | Score: | **3/5** |  |  |  |
| **Filippi** | **Screening questions (all types)** | S1. Are there clear research questions? | Yes |  |  |  |
|  |  | S2. Do the collected data allow to address the research questions? | Yes |  |  |  |
|  | **3. Quantative non-randomized**  **Analytical cross-sectional study** | 3.1. Are all the participants representative to the target population? |  |  | Can’t tell | Limited data on patient group, i.e. no information on gender or which diagnostic criteria were used. |
|  |  | 3.2. Are the measurements appropriate regarding both the outcome and intervention (or exposure?) | Yes |  |  |  |
|  |  | 3.3. Are there complete outcome data? | Yes |  |  |  |
|  |  | 3.4. Are the confounders accounted for in the design and analysis? | Yes |  |  |  |
|  |  | 3.5. During the study period, is the intervention administered (or exposure occurred) as intended? | Yes |  |  |  |
|  | **Total** | Score: | **4/5** |  |  |  |
| **Giovannoni** | **Screening questions (all types)** | S1. Are there clear research questions? | Yes |  |  |  |
|  |  | S2. Do the collected data allow to address the research questions? | Yes |  |  |  |
|  | **2. Quantitative randomized controlled trials** | 2.1. Is randomization appropriately performed? | Yes |  |  |  |
|  |  | 2.2 Are the groups comparable at baseline? | Yes |  |  |  |
|  |  | 2.3 Are there complete outcome data? | Yes |  |  | The study ended prematurely, but for the analyses at week 48 analyses there were sufficient data on >70% of included patients. |
|  |  | 2.4. Are outcome assessors blinded to the intervention provided? | Yes |  |  |  |
|  |  | 2.5. Did the participants adhere to the assigned intervention? | Yes |  |  |  |
|  | **Total** | Score: | **5/5** |  |  |  |
| **Harding** | **Screening questions (all types)** | S1. Are there clear research questions? | Yes |  |  |  |
|  |  | S2. Do the collected data allow to address the research questions? | Yes |  |  |  |
|  | **3. Quantative non-randomized**  **Cohort** | 3.1. Are all the participants representative to the target population? | Yes |  |  |  |
|  |  | 3.2. Are the measurements appropriate regarding both the outcome and intervention (or exposure?) |  | No |  | The measurements of clinical outcomes were only partly standardized. |
|  |  | 3.3. Are there complete outcome data? | Yes |  |  |  |
|  |  | 3.4. Are the confounders accounted for in the design and analysis? | Yes |  |  |  |
|  |  | 3.5. During the study period, is the intervention administered (or exposure occurred) as intended? | Yes |  |  |  |
|  | **Total** | Score: | **4/5** |  |  |  |
| **Hawker** | **Screening questions (all types)** | S1. Are there clear research questions? | Yes |  |  |  |
|  |  | S2. Do the collected data allow to address the research questions? | Yes |  |  |  |
|  | **2. Quantitative randomized controlled trials** | 2.1. Is randomization appropriately performed? |  |  | Can’t tell | Method of randomization was insufficiently described. |
|  |  | 2.2 Are the groups comparable at baseline? | Yes |  |  |  |
|  |  | 2.3 Are there complete outcome data? | Yes |  |  |  |
|  |  | 2.4. Are outcome assessors blinded to the intervention provided? | Yes |  |  |  |
|  |  | 2.5. Did the participants adhere to the assigned intervention? | Yes |  |  |  |
|  | **Total** | Score: | **4/5** |  |  |  |
| **Hughes** | **Screening questions (all types)** | S1. Are there clear research questions? | Yes |  |  |  |
|  |  | S2. Do the collected data allow to address the research questions? | Yes |  |  |  |
|  | **3. Quantative non-randomized**  **Real-world, registry** | 3.1. Are all the participants representative to the target population? | Yes |  |  |  |
|  |  | 3.2. Are the measurements appropriate regarding both the outcome and intervention (or exposure?) |  |  | Can’t tell | The was no clearly standardized clinical and radiological follow-up. |
|  |  | 3.3. Are there complete outcome data? | Yes |  |  |  |
|  |  | 3.4. Are the confounders accounted for in the design and analysis? | Yes |  |  |  |
|  |  | 3.5. During the study period, is the intervention administered (or exposure occurred) as intended? | Yes |  |  |  |
|  | **Total** | Score: | **4/5** |  |  |  |
| **Khaleeli** | **Screening questions (all types)** | S1. Are there clear research questions? | Yes |  |  |  |
|  |  | S2. Do the collected data allow to address the research questions? | Yes |  |  |  |
|  | **3. Quantative non-randomized**  **Cohort** | 3.1. Are all the participants representative to the target population? | Yes |  |  |  |
|  |  | 3.2. Are the measurements appropriate regarding both the outcome and intervention (or exposure?) | Yes |  |  | It is unclear if EDSS raters were blind to MRI outcomes, but otherwise clear and appropriate measures. |
|  |  | 3.3. Are there complete outcome data? |  |  | Can’t tell | Only 19/45 patients received the MRI at 5 year follow-up. |
|  |  | 3.4. Are the confounders accounted for in the design and analysis? | Yes |  |  |  |
|  |  | 3.5. During the study period, is the intervention administered (or exposure occurred) as intended? | Yes |  |  |  |
|  | **Total** | Score: | **4/5** |  |  |  |
| **Kidd** | **Screening questions (all types)** | S1. Are there clear research questions? | Yes |  |  |  |
|  |  | S2. Do the collected data allow to address the research questions? | Yes |  |  |  |
|  | **3. Quantative non-randomized**  **Cohort** | 3.1. Are all the participants representative to the target population? |  | No |  | All relapses excluded were excluded. There were no data given on sex. The included patients were relatively young age at onset (31 years). |
|  |  | 3.2. Are the measurements appropriate regarding both the outcome and intervention (or exposure?) | Yes |  |  |  |
|  |  | 3.3. Are there complete outcome data? | Yes |  |  |  |
|  |  | 3.4. Are the confounders accounted for in the design and analysis? | Yes |  |  |  |
|  |  | 3.5. During the study period, is the intervention administered (or exposure occurred) as intended? | Yes |  |  |  |
|  | **Total** | Score: | **4/5** |  |  |  |
| **Kremenchutzky** | **Screening questions (all types)** | S1. Are there clear research questions? | Yes |  |  |  |
|  |  | S2. Do the collected data allow to address the research questions? | Yes |  |  |  |
|  | **3. Quantative non-randomized**  **Real-world, registry** | 3.1. Are all the participants representative to the target population? | Yes |  |  |  |
|  |  | 3.2. Are the measurements appropriate regarding both the outcome and intervention (or exposure?) |  | No |  | There was no standardized follow-up, and no clear definition of relapse. |
|  |  | 3.3. Are there complete outcome data? | Yes |  |  |  |
|  |  | 3.4. Are the confounders accounted for in the design and analysis? | Yes |  |  |  |
|  |  | 3.5. During the study period, is the intervention administered (or exposure occurred) as intended? | Yes |  |  |  |
|  | **Total** | Score: | **4/5** |  |  |  |
| **Leary** | **Screening questions (all types)** | S1. Are there clear research questions? | Yes |  |  |  |
|  |  | S2. Do the collected data allow to address the research questions? | Yes |  |  |  |
|  | **2. Quantitative randomized controlled trials** | 2.1. Is randomization appropriately performed? | Yes |  |  | All relapses were excluded. |
|  |  | 2.2 Are the groups comparable at baseline? | Yes |  |  |  |
|  |  | 2.3 Are there complete outcome data? | Yes |  |  |  |
|  |  | 2.4. Are outcome assessors blinded to the intervention provided? | Yes |  |  |  |
|  |  | 2.5. Did the participants adhere to the assigned intervention? |  | No |  | Seven of fifteen patients on interferon 60 had a dose reduction. |
|  | **Total** | Score: | **4/5** |  |  |  |
| **Lorscheider** | **Screening questions (all types)** | S1. Are there clear research questions? | Yes |  |  |  |
|  |  | S2. Do the collected data allow to address the research questions? | Yes |  |  |  |
|  | **3. Quantative non-randomized**  **Real-world, registry** | 3.1. Are all the participants representative to the target population? | Yes |  |  |  |
|  |  | 3.2. Are the measurements appropriate regarding both the outcome and intervention (or exposure?) |  |  | Can’t tell | There was no standardized follow-up. |
|  |  | 3.3. Are there complete outcome data? |  | No |  | There were many missing MRI data. |
|  |  | 3.4. Are the confounders accounted for in the design and analysis? |  | No |  | Groups were propensity matched, but the treated group still showed more disease activity. |
|  |  | 3.5. During the study period, is the intervention administered (or exposure occurred) as intended? | Yes |  |  |  |
|  | **Total** | Score: | **2/5** |  |  |  |
| **Lublin** | **Screening questions (all types)** | S1. Are there clear research questions? | Yes |  |  |  |
|  |  | S2. Do the collected data allow to address the research questions? | Yes |  |  |  |
|  | **2. Quantitative randomized controlled trials** | 2.1. Is randomization appropriately performed? | Yes |  |  |  |
|  |  | 2.2 Are the groups comparable at baseline? | Yes |  |  |  |
|  |  | 2.3 Are there complete outcome data? | Yes |  |  |  |
|  |  | 2.4. Are outcome assessors blinded to the intervention provided? | Yes |  |  |  |
|  |  | 2.5. Did the participants adhere to the assigned intervention? |  | No |  | About a third of patients in treatment groups discontinued treatment. MRI protocol was unspecified. |
|  | **Total** | Score: | **4/5** |  |  |  |
| **Lycklama a Nijeholt** | **Screening questions (all types)** | S1. Are there clear research questions? | Yes |  |  |  |
|  |  | S2. Do the collected data allow to address the research questions? | Yes |  |  |  |
|  | **3. Quantative non-randomized**  **Cross-sectional analytical** | 3.1. Are all the participants representative to the target population? | Yes |  |  |  |
|  |  | 3.2. Are the measurements appropriate regarding both the outcome and intervention (or exposure?) | Yes |  |  |  |
|  |  | 3.3. Are there complete outcome data? | Yes |  |  |  |
|  |  | 3.4. Are the confounders accounted for in the design and analysis? | Yes |  |  | There are only correlations are described, therefore: not applicable. |
|  |  | 3.5. During the study period, is the intervention administered (or exposure occurred) as intended? | Yes |  |  |  |
|  | **Total** | Score: | **5/5** |  |  |  |
| **Mateo Paz Soldan** | **Screening questions (all types)** | S1. Are there clear research questions? | Yes |  |  |  |
|  |  | S2. Do the collected data allow to address the research questions? | Yes |  |  |  |
|  | **3. Quantative non-randomized**  **Cohort** | 3.1. Are all the participants representative to the target population? | Yes |  |  |  |
|  |  | 3.2. Are the measurements appropriate regarding both the outcome and intervention (or exposure?) |  | No |  | There was no clear definition of relapses and no clearly standardized follow-up. |
|  |  | 3.3. Are there complete outcome data? | Yes |  |  |  |
|  |  | 3.4. Are the confounders accounted for in the design and analysis? | Yes |  |  |  |
|  |  | 3.5. During the study period, is the intervention administered (or exposure occurred) as intended? | Yes |  |  |  |
|  | **Total** | Score: | **4/5** |  |  |  |
| **Montalban** | **Screening questions (all types)** | S1. Are there clear research questions? | Yes |  |  |  |
|  |  | S2. Do the collected data allow to address the research questions? | Yes |  |  |  |
|  | **2. Quantitative randomized controlled trials** | 2.1. Is randomization appropriately performed? | Yes |  |  |  |
|  |  | 2.2 Are the groups comparable at baseline? | Yes |  |  |  |
|  |  | 2.3 Are there complete outcome data? | Yes |  |  |  |
|  |  | 2.4. Are outcome assessors blinded to the intervention provided? | Yes |  |  |  |
|  |  | 2.5. Did the participants adhere to the assigned intervention? | Yes |  |  |  |
|  | **Total** | Score: | **5/5** |  |  |  |
| **Naser Moghadasi** | **Screening questions (all types)** | S1. Are there clear research questions? | Yes |  |  |  |
|  |  | S2. Do the collected data allow to address the research questions? | Yes |  |  |  |
|  | **3. Quantative non-randomized**  **Before-and-after study** | 3.1. Are all the participants representative to the target population? | Yes |  |  |  |
|  |  | 3.2. Are the measurements appropriate regarding both the outcome and intervention (or exposure?) |  | No |  | The radiological and clinical outcome measures were only partly standardized. |
|  |  | 3.3. Are there complete outcome data? | Yes |  |  |  |
|  |  | 3.4. Are the confounders accounted for in the design and analysis? |  | No |  | There was no control group; so no correction for placebo-effect or natural history. |
|  |  | 3.5. During the study period, is the intervention administered (or exposure occurred) as intended? | Yes |  |  |  |
|  | **Total** | Score: | **3/5** |  |  |  |
| **Perez-Miralles** | **Screening questions (all types)** | S1. Are there clear research questions? | Yes |  |  |  |
|  |  | S2. Do the collected data allow to address the research questions? | Yes |  |  |  |
|  | **3. Quantative non-randomized**  **Cohort** | 3.1. Are all the participants representative to the target population? | Yes |  |  |  |
|  |  | 3.2. Are the measurements appropriate regarding both the outcome and intervention (or exposure?) |  | No |  | The clinical and radiological outcome measures were only partly standardized. |
|  |  | 3.3. Are there complete outcome data? | Yes |  |  |  |
|  |  | 3.4. Are the confounders accounted for in the design and analysis? | Yes |  |  |  |
|  |  | 3.5. During the study period, is the intervention administered (or exposure occurred) as intended? | Yes |  |  |  |
|  | **Total** | Score: | **4/5** |  |  |  |
| **Petrou** | **Screening questions (all types)** | S1. Are there clear research questions? | Yes |  |  |  |
|  |  | S2. Do the collected data allow to address the research questions? | Yes |  |  |  |
|  | **2. Quantitative randomized controlled trials** | 2.1. Is randomization appropriately performed? |  |  | Can’t tell | The method of randomization was not described. |
|  |  | 2.2 Are the groups comparable at baseline? | Yes |  |  |  |
|  |  | 2.3 Are there complete outcome data? | Yes |  |  |  |
|  |  | 2.4. Are outcome assessors blinded to the intervention provided? | Yes |  |  |  |
|  |  | 2.5. Did the participants adhere to the assigned intervention? | Yes |  |  |  |
|  | **Total** | Score: | **4/5** |  |  |  |
| **Pohlau** | **Screening questions (all types)** | S1. Are there clear research questions? |  |  |  |  |
|  |  | S2. Do the collected data allow to address the research questions? |  |  |  |  |
|  | **2. Quantitative randomized controlled trials** | 2.1. Is randomization appropriately performed? | Yes |  |  |  |
|  |  | 2.2 Are the groups comparable at baseline? | Yes |  |  |  |
|  |  | 2.3 Are there complete outcome data? |  | No |  | Only 49% of patients completed the 112 weeks of study. |
|  |  | 2.4. Are outcome assessors blinded to the intervention provided? |  |  | Can’t tell | There was an incomplete description of blinding procedures. |
|  |  | 2.5. Did the participants adhere to the assigned intervention? | Yes |  |  |  |
|  | **Total** |  | **3/5** |  |  |  |
| **Ratzer** | **Screening questions (all types)** | S1. Are there clear research questions? | Yes |  |  |  |
|  |  | S2. Do the collected data allow to address the research questions? | Yes |  |  |  |
|  | **3. Quantative non-randomized**  **Before-and-after study** | 3.1. Are all the participants representative to the target population? |  | No |  | It was unclear which diagnostic criteria were used. |
|  |  | 3.2. Are the measurements appropriate regarding both the outcome and intervention (or exposure?) | Yes |  |  |  |
|  |  | 3.3. Are there complete outcome data? | Yes |  |  |  |
|  |  | 3.4. Are the confounders accounted for in the design and analysis? |  | No |  | There was no control group. |
|  |  | 3.5. During the study period, is the intervention administered (or exposure occurred) as intended? | Yes |  |  |  |
|  | **Total** | Score: | **3/5** |  |  |  |
| **Romme-Christensen** | **Screening questions (all types)** | S1. Are there clear research questions? | Yes |  |  |  |
|  |  | S2. Do the collected data allow to address the research questions? | Yes |  |  |  |
|  | **3. Quantative non-randomized**  **Cohort** | 3.1. Are all the participants representative to the target population? | Yes |  |  |  |
|  |  | 3.2. Are the measurements appropriate regarding both the outcome and intervention (or exposure?) | Yes |  |  |  |
|  |  | 3.3. Are there complete outcome data? |  | No |  | There was a dropout of 23.3% in the study. |
|  |  | 3.4. Are the confounders accounted for in the design and analysis? |  | No |  | There was no control group. |
|  |  | 3.5. During the study period, is the intervention administered (or exposure occurred) as intended? | Yes |  |  |  |
|  | **Total** | Score: | **3/5** |  |  |  |
| **Salzer** | **Screening questions (all types)** | S1. Are there clear research questions? | Yes |  |  |  |
|  |  | S2. Do the collected data allow to address the research questions? | Yes |  |  | For the question concerning safety: yes |
|  | **3. Quantative non-randomized**  **Before-and-after study** | 3.1. Are all the participants representative to the target population? |  | No |  | It was unclear which diagnostic criteria were used. |
|  |  | 3.2. Are the measurements appropriate regarding both the outcome and intervention (or exposure?) |  | No |  | There was no standardized follow-up. |
|  |  | 3.3. Are there complete outcome data? | Yes |  |  |  |
|  |  | 3.4. Are the confounders accounted for in the design and analysis? | Yes |  |  |  |
|  |  | 3.5. During the study period, is the intervention administered (or exposure occurred) as intended? | Yes |  |  |  |
|  | **Total** | Score: | **3/5** |  |  |  |
| **Sastre-Garriga** | **Screening questions (all types)** | S1. Are there clear research questions? | Yes |  |  |  |
|  |  | S2. Do the collected data allow to address the research questions? | Yes |  |  |  |
|  | **4. Quantitative descriptive : Case-series** | 4.1. Is the sampling strategy relevant to address the research question? | Yes |  |  |  |
|  |  | 4.2. Is the sample representative of the target population? | Yes |  |  |  |
|  |  | 4.3. Are the measurements appropriate? | Yes |  |  |  |
|  |  | 4.4. Is the risk of nonresponse bias low? | Yes |  |  |  |
|  |  | 4.5. Is the statistical analysis appropriate to answer the research question? | Yes |  |  |  |
|  | **Total** | Score: | **5/5** |  |  |  |
| **Thompson** | **Screening questions (all types)** | S1. Are there clear research questions? | Yes |  |  |  |
|  |  | S2. Do the collected data allow to address the research questions? | Yes |  |  |  |
|  | **3. Quantative non-randomized**  **Time-series** | 3.1. Are all the participants representative to the target population? |  | No |  | All relapses (also after progression) were excluded. |
|  |  | 3.2. Are the measurements appropriate regarding both the outcome and intervention (or exposure?) | Yes |  |  |  |
|  |  | 3.3. Are there complete outcome data? | Yes |  |  |  |
|  |  | 3.4. Are the confounders accounted for in the design and analysis? | Yes |  |  |  |
|  |  | 3.5. During the study period, is the intervention administered (or exposure occurred) as intended? | Yes |  |  |  |
|  | **Total** | Score: | **4/5** |  |  |  |
| **Wolinsky** | **Screening questions (all types)** | S1. Are there clear research questions? | Yes |  |  |  |
|  |  | S2. Do the collected data allow to address the research questions? | Yes |  |  |  |
|  | **2. Quantitative randomized controlled trials** | 2.1. Is randomization appropiately performed? |  |  | Can’t tell | Method of randomization was insufficiently described. |
|  |  | 2.2 Are the groups comparable at baseline? | Yes |  |  |  |
|  |  | 2.3 Are there complete outcome data? | Yes |  |  |  |
|  |  | 2.4. Are outcome assessors blinded to the intervention provided? | Yes |  |  |  |
|  |  | 2.5. Did the participants adhere to the assigned intervention? | Yes |  |  |  |
|  | **Total** | Score: | **4/5** |  |  |  |
| **Zephir** | **Screening questions (all types)** | S1. Are there clear research questions? | Yes |  |  |  |
|  |  | S2. Do the collected data allow to address the research questions? | Yes |  |  |  |
|  | **3. Quantative non-randomized**  **Cohort** | 3.1. Are all the participants representative to the target population? | Yes |  |  |  |
|  |  | 3.2. Are the measurements appropriate regarding both the outcome and intervention (or exposure?) |  | No |  | No clear definition of a relapse was given. |
|  |  | 3.3. Are there complete outcome data? | Yes |  |  |  |
|  |  | 3.4. Are the confounders accounted for in the design and analysis? | Yes |  |  |  |
|  |  | 3.5. During the study period, is the intervention administered (or exposure occurred) as intended? | Yes |  |  |  |
|  | **Total** | Score: | **4/5** |  |  |  |
| **Zecca** | **Screening questions (all types)** | S1. Are there clear research questions? | Yes |  |  |  |
|  |  | S2. Do the collected data allow to address the research questions? | Yes |  |  | For the safety question: yes. For the efficacy question: no. |
|  | **3. Quantative non-randomized**  **Cohort** | 3.1. Are all the participants representative to the target population? | Yes |  |  |  |
|  |  | 3.2. Are the measurements appropriate regarding both the outcome and intervention (or exposure?) | Yes |  |  |  |
|  |  | 3.3. Are there complete outcome data? |  | No |  | There was no information on 96 patients without follow-up after induction; only 78.7% of cohort with completed follow-up (<80%). |
|  |  | 3.4. Are the confounders accounted for in the design and analysis? | Yes |  |  |  |
|  |  | 3.5. During the study period, is the intervention administered (or exposure occurred) as intended? | Yes |  |  |  |
|  | **Total** | Score: | **4/5** |  |  |  |
